# The origin and remolding of genomic islands of differentiation in the European sea bass

**DOI:** 10.1038/s41467-018-04963-6

**Published:** 2018-06-28

**Authors:** Maud Duranton, François Allal, Christelle Fraïsse, Nicolas Bierne, François Bonhomme, Pierre-Alexandre Gagnaire

**Affiliations:** 1 Institut des Sciences de l’Evolution de Montpellier - UMR5554 UM-CNRS-IRD-EPHE, Place Eugène Bataillon, 34095 Montpellier, France; 2 Université de Montpellier, Place Eugène Bataillon, 34095 Montpellier, France; 3 MARBEC, Université de Montpellier, Ifremer-CNRS-IRD-UM, 34250 Palavas-les-Flots, France

## Abstract

Speciation is a complex process that leads to the progressive establishment of reproductive isolation barriers between diverging populations. Genome-wide comparisons between closely related species have revealed the existence of heterogeneous divergence patterns, dominated by genomic islands of increased divergence supposed to contain reproductive isolation loci. However, this divergence landscape only provides a static picture of the dynamic process of speciation, during which confounding mechanisms unrelated to speciation can interfere. Here we use haplotype-resolved whole-genome sequences to identify the mechanisms responsible for the formation of genomic islands between Atlantic and Mediterranean sea bass lineages. Local ancestry patterns show that genomic islands first emerged in allopatry through linked selection acting on a heterogeneous recombination landscape. Then, upon secondary contact, preexisting islands were strongly remolded by differential introgression, revealing variable fitness effects among regions involved in reproductive isolation. Interestingly, we find that divergent regions containing ancient polymorphisms conferred the strongest resistance to introgression.

## Abstract (web summary)

The speciation process tends to generate ‘genomic islands’ of increased divergence. Here, the authors use haplotype–resolved whole-genome sequences of European sea bass lineages to infer divergence history and show that linked selection generated genomic islands that resist introgression at secondary contact.

## Introduction

Understanding how genetic differences accumulate between populations over time to eventually form new species is one of the main objectives in evolutionary biology^1,2^. Speciation is generally thought as a gradual mechanism that proceeds through intermediate stages whereby gene flow is not completely interrupted and genomes remain permeable to genetic exchanges^3–6^. As long as species can still hybridize, studying gene exchange provides access to variety of evolutionary mechanisms involved at different stages of the speciation process^7,8^. Advanced sequencing technologies now provide a genome-wide view of divergence between closely related species, improving our understanding of how speciation unfolds at the molecular level^9^. A growing number of speciation genomics studies have demonstrated the existence of heterogeneous genomic divergence patterns between entities at different stages of speciation^10–20^. However, difficulties to relate empirical divergence patterns to the underlying mechanisms involved in their formation limit the potential of speciation genomics approaches^21,22^.

Heterogeneous genome divergence between taxa can have several possible causes that need to be individually assessed for understanding the underlying mechanisms generating regions of increased divergence^23,24^, the so-called genomic islands^10,11,20^. Among them, accelerated rates of lineage sorting within populations due to recurrent events of either selective sweeps^25^ or background selection (BGS)^26^ can generate incidental islands of relative divergence that are not necessarily related to reproductive isolation (RI)^27^. An important objective of speciation research is therefore to identify and understand the origin of genomic islands associated with barrier loci responsible for gene flow reduction between diverging populations^22^. Such islands may be themselves explained by different mechanisms depending on the intensity and timing of gene flow and the genomic architecture of RI^24^. Elucidating the typical conditions under which each mechanism is at play is central to understanding the roles of selection and gene flow in the speciation process.

The identification of genomic regions that are truly resistant to introgression remains a challenging task, especially because the aforementioned mechanisms are influenced by the recombination landscape and therefore tend to affect similar regions of the genome^27–29^. To disentangle the role of these confounding factors, substantial levels of gene flow may be needed to properly reveal the genomic regions involved in RI. Moreover, the analysis of gene flow and selection may be facilitated by the direct detection of introgressed chromosomal segments, combined with an explicit consideration of the demographic history^30^. Here we developed this type of approach in a high gene flow marine species.

The European sea bass (*Dicentrarchus labrax*) provides an interesting model to understand the evolution of genomic islands^31^. The species is subdivided into an Atlantic and a Mediterranean lineage that hybridize in the Alboran sea^32^. Historical demographic inferences revealed that the two lineages have started to diverge in allopatry around 300,000 years before present (BP) and then experienced a post-glacial secondary contact generating varying rates of introgression across the genome^31^. This evolutionary history mirrors the distributional range shifts that occurred across many taxa during glacial periods especially in the Atlantic–Mediterranean region^33^, which are recognized as an important source of species diversification^34,35^. Admittedly, however, the divergence history of sea bass lineages may involve a more complex succession of divergence and contact periods potentially paced by the quasi-100,000-year glacial cycles during the Pleistocene^36^. Here we characterize local ancestry patterns using haplotype-resolved whole-genome sequences within a geographic context to (i) infer the divergence history of sea bass lineages from the length spectrum of introgressed tracts and shared haplotypes and (ii) identify the different mechanisms involved in the formation and remolding of genomic islands of differentiation. Our results show that genomic regions experiencing stronger linked selection due to low recombination not only diverge faster during allopatric episodes but also better resist introgression in the presence of gene flow. These findings support that multiple loci affect RI in sea bass, with the most divergent having the strongest effects.

## Results

### Spatial population structure and admixture

**Fig. 1:**
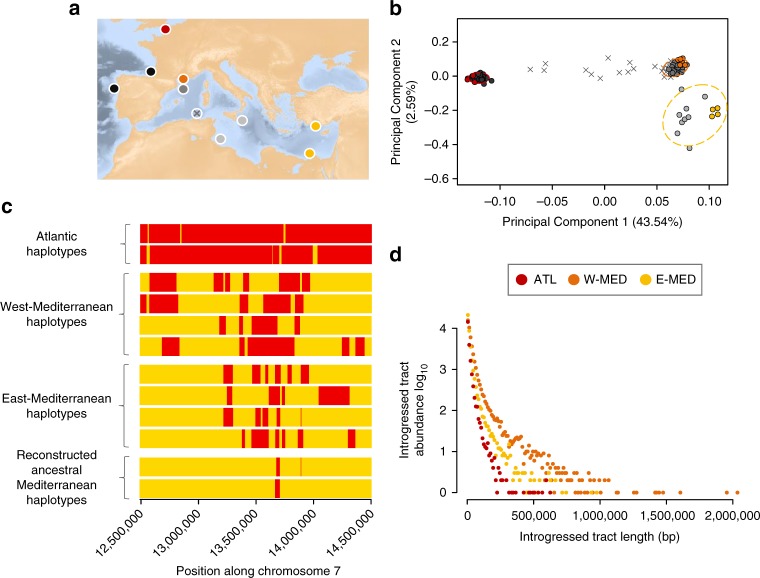
Spatial population structure and local ancestry patterns. **a** Geographical location of samples, including the newly sequenced genomes (colored circles) and additional reference samples from the Atlantic (dark gray), W-MED (gray), E-MED (light gray), and the Algerian admixture zone (gray crosses). **b** Principal Component Analysis of newly sequenced genomes combined with 112 individuals genotyped at 13,094 common SNPs (MAF > 0.1). The first PCA axis distinguishes Atlantic and Mediterranean populations while the second axis reveals a subtle population structure between W-MED and E-MED. Some individuals from the Algerian coast represent admixed genotypes between Atlantic and Mediterranean populations. **c** Schematic representation of a 2 Mb region within chromosome 7, showing the mosaic of ancestry blocks derived from Atlantic (red) and Mediterranean (yellow) populations. For simplification, we only display two individual haplotypes from Atlantic samples, four from W-MED, four from E-MED, and two reconstructed ancestral Mediterranean haplotypes from E-MED samples. **d** Migrant tract length distribution obtained for the Atlantic (red, showing tracts of Mediterranean origin), W-MED (orange), and E-MED (yellow) populations (showing tracts of Atlantic origin) using four individuals per population and a total of 2,628,725 phased SNPs

The genetic relationships of the newly sequenced genomes with respect to the range-wide population structure of the European sea bass were evaluated with a Principal Component (PC) Analysis including 112 additional individuals genotyped at 13,094 common single nucleotide polymorphisms (SNPs; Fig. 1a, b and Supplementary Note 2). The main component of genetic variation (axis 1, 43.54% of explained variance) clearly distinguished Atlantic from Mediterranean populations, while the second axis (2.59%) revealed a subtle genetic differentiation between eastern and western Mediterranean basins (E-MED and W-MED, respectively). Genetic admixture was found to occur along the Algerian coast, which is the principal zone where Atlantic alleles enter the Mediterranean sea. The resulting inflow of Atlantic alleles within the Mediterranean generates a longitudinal gradient of introgression illustrated by a shift between W-MED and E-MED samples along the first PC axis (Fig. 1b).

### Migrant tracts' identification

Spatial introgression patterns were also detected at the chromosome level using local ancestry inference based on 2,628,725 phased SNPs. The proportion of the genome occupied by migrant tracts was twice higher in the W-MED (31%) compared to the E-MED population (13%), which also displayed shorter migrant tracts (Fig. 1c, d). The longest introgressed haplotype detected in the W-MED (2.03 Mb) was twice as long as the longest one found in the E-MED population (0.98 Mb). More generally, the genome-wide distribution of migrant tract length showed a reduced abundance of tracts over all length classes in the E-MED compared to the W-MED population. This shift is consistent with the action of recombination that progressively erodes recently introgressed tracts as they diffuse by migration from the entrance to the bottom of the Mediterranean sea. Consistently, this effect was not apparent for the shortest migrant tracts (i.e., <50 kb) that probably reside in the Mediterranean sea for a much longer time than the time needed to diffuse from west to east. The Atlantic population was the least introgressed, with <5% of its genome occupied by tracts of Mediterranean ancestry. Migrant tracts were also shorter (maximum length 0.62 Mb) and less abundant over the whole-length spectrum (Fig. 1c, d). This is consistent with a reduced amount of gene flow from the Mediterranean to the Atlantic population^31^. Finally, our method for reconstructing ancestral Mediterranean genomes effectively removed migrant tracts (Supplementary Note 4), leaving very small residual amounts of Atlantic haplotypes in the reconstructed Mediterranean genomes (Fig. 1c), except for genomic regions that were strongly introgressed by Atlantic alleles (Supplementary Figs. 5 and 6).

### Analysis of migrant tract length distribution

**Fig. 2:**
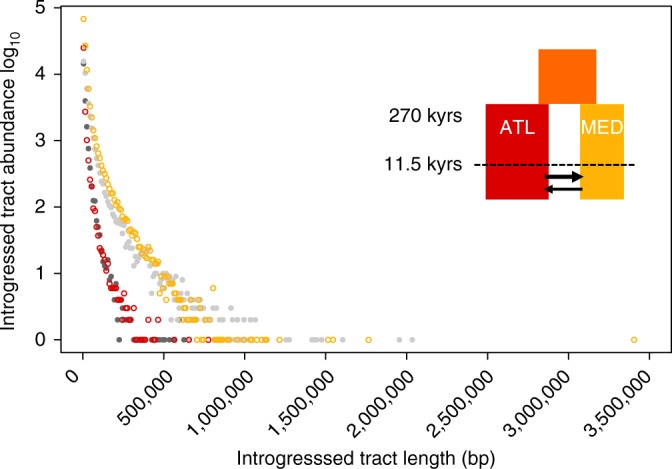
Observed and simulated migrant tract length distributions in the Atlantic and Mediterranean populations. Observed distributions (gray) of migrant tract length are compared with simulated distributions (colored) under the post-glacial secondary contact scenario illustrated in the top-right corner^31^. The abundance of introgressed tracts as a function of their length is represented for observed vs. simulated data in the Atlantic (dark gray vs. red circles, showing tracts of Mediterranean origin) and Mediterranean populations (light gray vs. yellow circles, showing tracts of Atlantic origin)

An 85% fraction of the 100 kb windows located in low-recombining regions of the Mediterranean genomes present introgressed Atlantic tracts that are on average >50 kb (Supplementary Fig. 7). Using a recombination clock, we found that this observation is consistent with introgression occurring during the past 17,000 years. Since the most part of the introgressed tracts length distribution is consistent with a recent secondary contact, we evaluated the goodness-of-fit of the previously inferred post-glacial secondary contact model^31^, which places the onset of gene flow 11,500 years ago. We performed coalescent simulations with variable recombination rates under this model to generate whole-genome data using the recombination structure of sea bass chromosomes. The length distribution of migrant tracts obtained from these simulations reproduced well the observed distribution for both the Atlantic and Mediterranean populations (Fig. 2). Therefore, the secondary contact model inferred from the joint site frequency spectrum without using linkage information^31^ has a high predictive power regarding the length distribution of introgressed tracts when accounting for recombination rate variation.

### Testing waves of historical gene flow

**Fig. 3:**
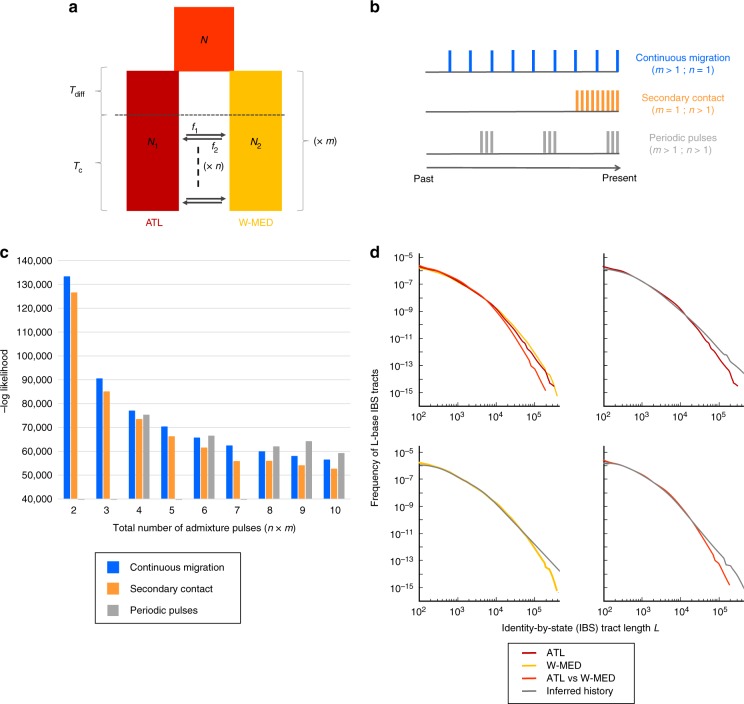
Demographic history inferred from the length distribution of IBS tracts. **a** Flexible demographic model accounting for multiple equal-length episodes of divergence and gene flow between Atlantic and Mediterranean sea bass populations. An ancestral population of size *N* splits into two populations of size *N*_1_ and *N*_2_, experiencing one to several (*m*) cycles of interrupted gene flow during *T*_diff_ generations followed by migration during *T*_c_ generations. Each contact episode contains one to several (*n*) pulses of admixture (black arrows), replacing Mediterranean and Atlantic populations by a proportion *f*_1_ and *f*_2_ of migrants, respectively. The most recent admixture pulse occurs at time *T*_c_/100 before the end of each contact episode, and preceding pulses are homogeneously distributed every (*T*_c_−*T*_c_/100)/*n* time interval. **b** Modeling scenarios fall into three different categories according to the distribution of admixture pulses over time: continuous migration, secondary contact, and periodic pulses. The illustrated example shows these three categories with nine pulses represented by vertical bars along the timeline. **c** The log-likelihood values obtained for each of the three modeling scenarios from two to ten admixture pulses. Two possible configurations of the periodic pulses scenario exist for models including a total of six, eight, and ten pulses (Supplementary Table 2), only the best of which is represented here. The periodic pulses scenario is not defined for two, three, five, and seven pulses. **d** Goodness-of-fit of the best model (*m* = 1, *n* = 10), showing the length distributions of IBS tracts from observed data (colored lines) compared to model prediction (gray lines). Upper-left: the three distributions observed within ATL and W-MED and between ATL and W-MED populations. Upper-right: model fit within ATL. Lower-left: model fit within W-MED. Lower-right: model fit between ATL and W-MED

Initial divergence between sea bass lineages has been dated around 270,000 years BP^31^, corresponding to three glacial cycles^36^ during which possible genetic interactions may have occurred when interglacial conditions were similar to present. In order to address whether short migrant tracts found within low-recombining regions of the genome could result from waves of historical gene flow, we developed a flexible model of divergence in which the history of admixture can take different forms (Fig. 3a). The modeling scenarios were subdivided into three categories according to the distribution of admixture pulses over time: (i) continuous migration, (ii) secondary contact, and (iii) periodic pulses (Fig. 3b). Our demographic inferences showed an increase in likelihood with increasing numbers of pulses in each scenario (Fig. 3c). This is because the total number of admixture pulses contained in each model (i.e., the product *m* × *n*) acts as a hidden nuisance parameter, even though the three different scenarios were built using the same number of model parameters. Therefore, we only compared likelihood values among scenarios with identical number of admixture pulses, considering up to 10 pulses in total (the likelihood tended to flatten out beyond this value). We found the secondary contact scenario to be the best-supported model across the entire range of admixture pulse number (Fig. 3c and Supplementary Table 2) and therefore found no support for a periodic pulse model with separate waves of gene flow. The best-fit secondary contact model (*n* *=* 10 pulses, Fig. 3d) provided clear support for asymmetric introgression, with a more than six-fold higher introgression rate from the Atlantic into the Mediterranean than in the opposite direction, which is consistent with previous findings^31^. The duration of allopatric divergence relative to the secondary contact period was found shorter than previously reported (Supplementary Table 3). Nevertheless, the splitting times estimated between Atlantic and Mediterranean sea bass lineages were highly consistent across methods (ca. 300,000 years BP for the best identity-by-state (IBS) tract model vs. 270,000 years BP from the best JAFS model^31^).

### Linking genomic islands to modes of selection

**Fig. 4:**
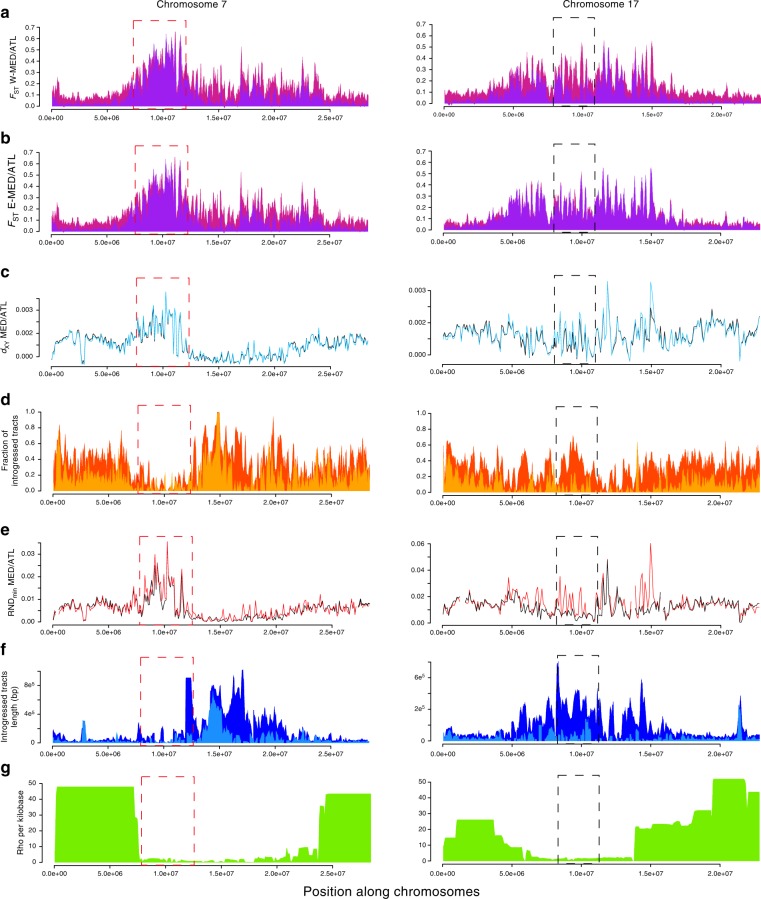
Population genetic statistics calculated in non-overlapping 100 kb windows along chromosomes 7 and 17. **a** *F*_ST_ measured between the Atlantic and contemporary (purple) or ancestral reconstructed (mauve) W-MED or E-MED (**b**) population. **c** *d*_XY_ calculated between the Atlantic and the W-MED (black) or E-MED (blue) population. **d** Fraction of introgressed tracts in the W-MED (orange) or E-MED (yellow) population. **e** RND_min_ measured between the Atlantic and W-MED (black) or E-MED (red) population. **f** Average length of introgressed tracts in the W-MED (dark blue) or E-MED (light blue) population. **g** Population-scaled recombination rate (*ρ* = 4*N*_e_*r*) averaged between Atlantic and Mediterranean population^31^

**Fig. 5:**
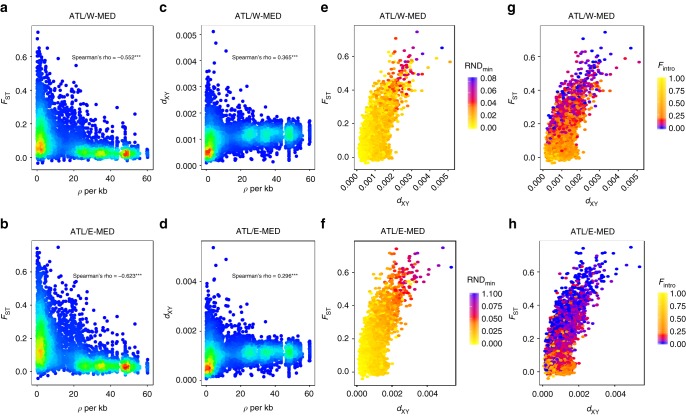
Relationships between divergence (*F*_ST_ and *d*_XY_), the population-scaled recombination rate (*ρ* = 4*N*_e_*r*), and introgression statistics (RND_min_ and *F*_intro_). **a**–**d** The density of points appears in color scale from low (blue) to high (red) densities. **e**–**h** The color scale indicates the value of RND_min_ (**e**, **f**) or the frequency of introgression (**g**, **h**) in the corresponding window from low (blue) to high (yellow) introgression rate values

We investigated chromosomal patterns of genetic differentiation (*F*_ST_) and absolute sequence divergence (*d*_XY_) between Atlantic and Mediterranean populations. We found highly varying levels of relative and absolute divergence across the genome, with Mb-scale regions of elevated divergence preferentially mapping to low-recombining regions (Fig. 4a–c and Supplementary Fig. 9). Consistent with predictions from the linked selection hypothesis^27,37^, *F*_ST_ and *d*_XY_ were, respectively, negatively and positively related to the population-scaled recombination rate (*ρ* = 4*N*_e_*r*) (Fig. 5a–d, Supplementary Figs. 10 and 11). However, the highest *F*_ST_ values tended to be associated with high values of *d*_XY_ mapping preferentially to low-recombining regions (Fig. 5), which is not expected under the single action of linked selection^38^. Specifically, we found that the most divergent windows in terms of both *F*_ST_ and *d*_XY_ were also associated with high RND_min_ values (Fig. 5e, f and Supplementary Fig. 12) or low frequencies of introgressed tracts (Fig. 5g, h and Supplementary Fig. 13), indicating increased resistance to gene flow. This is consistent with the similar levels of contemporary and ancestral *F*_ST_ values reconstructed in these regions (Fig. 4a, b, mauve vs. purple *F*_ST_ plots in the red dotted box).

**Fig. 6:**
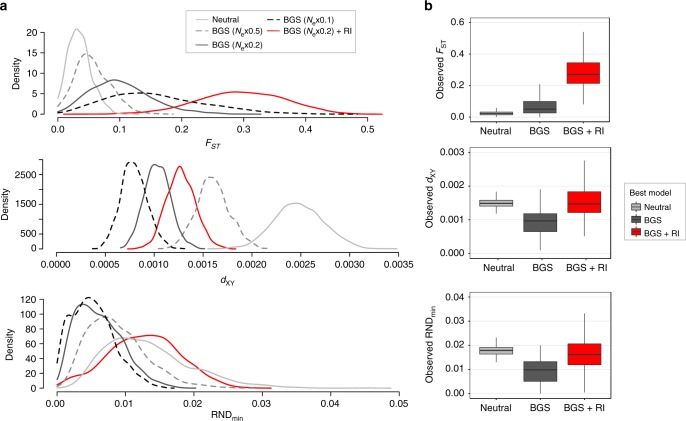
Simulations under different modes of selection to understand the mechanisms underlying genomic islands. **a** Comparison among simulated distributions of 100 kb window-averaged *F*_ST_ (top), *d*_XY_ (middle), and RND_min_ (bottom) obtained under different versions of the secondary contact scenario, including neutral divergence and introgression (Neutral), varying strengths of background selection (BGS, from 0.5 to 0.1 × *N*_e_), and BGS with reproductive isolation (BGS+RI). **b** Comparison among observed distributions of *F*_ST_ (top), *d*_XY_ (middle), and RND_min_ (bottom) for real genomic windows of 100 kb that were either assigned to the Neutral model (7% of windows), the BGS model (76% of windows), or the BGS+RI model (17% of windows) based on Euclidean distances to simulated data

In order to evaluate the extent to which these observations support the existence of genomic islands resistant to gene flow, we simulated the secondary contact scenario under different modes of selection. Our simulations show that only the BGS+RI model can reproduce the combinations of statistics observed within genomic islands characterized by high values of both *F*_ST_, *d*_XY_, and RND_min_ (Fig. 6a). By contrast, the Neutral model cannot generate high *F*_ST_ values due to migration, whereas, although the BGS model could reach such values provided sufficient strength of background selection (BGS), it would on the other hand generate low *d*_XY_ values. The comparison between observed and simulated data revealed that the BGS+RI model outperformed the Neutral and the BGS model for 16.7% of observed genomic windows, which are also characterized by high values of *d*_XY_, *F*_ST_, and RND_min_ (Fig. 6b). Therefore, barriers to gene flow between Atlantic and Mediterranean sea bass tend to involve regions of low recombination where both relative and absolute divergence are higher than expected under the sole effect of linked selection.

Variable degrees of resistance to gene flow among genomic regions were also detected using spatial comparisons of divergence patterns. Despite the strong correlation observed between ATL/W-MED and ATL/E-MED *F*_ST_ patterns (Supplementary Fig. 5), some peaks of ancestral differentiation almost completely vanished in ATL/W-MED contemporary patterns but remained remarkably unchanged in the ATL/E-MED comparison (Fig. 4, black dotted box). By contrast, some peaks seem to have resisted to introgression in both ATL/W-MED and ATL/E-MED comparisons (Fig. 4, red dotted box). These differences are consistent with variable strength of the barrier effect among genomic regions involved in RI and possibly to local introgression swamping (i.e., adaptive introgression in the W-MED or genetic incompatibilities that escaped from coupling).

Despite selection against introgression in some genomic regions, the length of introgressed tracts at the genome scale was largely determined by the local recombination rate (Fig. 4f–g). However, we found that the mean length of introgressed tracts was reduced (up to four-fold) in the close vicinity (<200 kb) of the strongest barriers to gene flow identified by outlier RND_min_ values (Supplementary Fig. 14), consistent with theoretical predictions^39^. When combined across all chromosomes, such regions only represented 4% of the genome.

Finally, we found a negative correlation between the local fraction of introgressed tracts and both ancestral *F*_ST_ and RND_max_ (Supplementary Fig. 15). This indicates that the regions with the highest level of precontact differentiation and maximal divergence were the less likely to introgress during the recent secondary contact episode. Moreover, observed values of *d*_XY_ and RND_min_ within genomic islands resistant to gene flow were higher than those obtained using simulations under the BGS+RI model. These results thus support that the haplotypes located in the genomic regions containing RI loci are older than the average age of alleles across the genome.

## Discussion

We used two different approaches based on haplotype information to resolve the history of divergence and gene flow between Atlantic and Mediterranean sea bass lineages. First, our coalescent simulations with recombination showed that the observed length distribution of introgressed tracts can be well reproduced by the post-glacial secondary contact model inferred in a previous study^31^. We then evaluated whether the presence of short introgressed tracts in low-recombining regions could indicate the existence of older admixture events. Although there is a possibility that, during the whole divergence history, the quasi-100,000-year glacial cycles^36^ have promoted discrete waves of gene flow, we did not find evidence for a cyclic connectivity model representing glacial oscillations. Instead, our demographic inferences based on the IBS tract spectrum also supported a scenario of secondary contact and confirmed that Atlantic and Mediterranean sea bass lineages have started to diverge around 300,000 years BP. We nonetheless detected an older time of contact that might explain the shortest introgressed tracts found in low-recombining regions.

Admittedly, the power of our inferences may be limited by the confounding effects of time and recombination on the length of introgressed tracts^40–42^. Therefore, haplotype-based methods may be sensitive to local inaccuracies in the estimation of recombination rate along the genome. Furthermore, although the magnitude of divergence was captured by the between-lineages spectrum of shared IBS tracts, the two within-lineages spectrums were highly similar, possibly leading to a lack of signal to precisely estimate the duration of secondary contact with the IBS tract method.

The two approaches implemented here neglect the effects of temporal changes in effective population size and selection against introgressed tracts. Populations surviving in glacial refugia possibly experienced bottlenecks^34^, which could have impacted the IBS tract spectrum^43^. Furthermore, the removal of long blocks of foreign ancestry by selection can reduce the average length of introgressed tracts, although we showed that this only happens in a minor fraction (4%) of the sea bass genome. Therefore, selection against migrant tracts is unlikely to cause significant violations to our neutral modeling approach, which should not interfere with its capacity to discriminate among alternative divergence scenarios. Nevertheless, unaccounted selection may explain the over-predicted abundance of introgressed tracts <500 kb in the Mediterranean (Fig. 2) and of IBS tracts >100 kb (Fig. 3). Future works will have to integrate the effect of selection against introgression within demographic models, as previously done for demographic inference from unphased data^6,44,45^.

The role of gene flow in generating genomic islands is a long standing debate^3,22,27,37,46,47^. In particular, whether gene flow simply remodels evolving or pre-existing divergence patterns or constrains divergence to evolve principally in low-recombining regions remains an open question^24^. Our results demonstrate that linked selection^48^ has increased the rate of lineage sorting in low-recombining regions, generating heterogeneous genome divergence between sea bass lineages during their geographic isolation. This was supported both by the genome-wide correlations between divergence and recombination and the reconstructed ancestral landscape of divergence. The reduction of recombination in the center of chromosomes relative to their peripheries is a common feature of fish genomes^49,50^, which is possibly due to crossover interference^51^ and male heterochiasmy^52^. Therefore, linked selection generating heterogeneous differentiation across the genome can be seen as a null expectation for allopatric divergence in sea bass, as in other teleost fish.

Gene flow after secondary contact has the potential to remodel heterogeneous divergence landscapes by eroding neutral differentiation^28^, sometimes rapidly^24^. However, how RI loci affect the dynamics of erosion of pre-existing islands of differentiation during secondary contact remains poorly understood. Our direct detection of introgressed tracts revealed broad variation in the rate of introgression among regions displaying similar levels of divergence. In some regions where ancestral peaks of *F*_ST_ have been found, the amount of introgression was high and the peaks almost vanished after secondary contact. This suggests that these incidental islands do not contain RI loci^27^, or if they contained any, have managed to escape coupling with other such genes. On the other hand, reduced introgression in many other regions confirms the view that introgressed alleles generally have negative fitness effects in the foreign genetic background^30,53^ (although we did not specifically address the extent of adaptive introgression in that study). The spatial comparison of introgression patterns between recipient populations (W-MED and E-MED) at variable distances from the source population (ATL) provides indirect cues about the distribution of fitness effects of introgressed tracts. Some genomic islands were resistant to introgression in both Mediterranean populations, indicating strong selection against introgressed tracts. In most genomic islands, however, introgression was more reduced in the E-MED compared to the W-MED. This either suggests that stronger migration overwhelms the effect of selection in the W-MED or that selection takes more time to remove weakly selected migrant tracts as they diffuse from the western to the eastern part of the Mediterranean sea.

An important result stemming from the analysis of introgression is that the degree of resistance to gene flow for a given region was positively related to the past level of differentiation and to the absolute divergence between haplotypes, independently of the local mutation rate. Therefore, the strength of selection against introgressed tracts at a given genomic location seems to be at least partly explained by the coalescence time between haplotypes. The expected amount of absolute divergence in low-recombining regions of the sea bass genome can be determined by summing values of ancestral diversity (*θ*_anc_ ≈ 0.001, estimated from the mean diversity of contemporary populations) and sequence divergence due to the accumulation of mutations in both lineages after split (2*μT* ≈ 0.001, determined using the divergence time estimated from demographic models). This amounts to *E*(*d*_XY_) = *θ*_anc_ + 2*μT* = 0.002, a value twice higher than the observed genome-wide average, which is decreased by introgression. By contrast, the strongest barriers to gene flow between Atlantic and Mediterranean sea bass involve regions with higher *d*_XY_ values ranging between 0.002 and 0.005. Our simulations under the BGS+RI model confirmed that this excess of coalescence time is not expected under a scenario whereby differential introgression reshapes a heterogeneous divergence landscape previously established by linked selection alone^38^. To explain these results, we thus need to consider the sorting of ancient polymorphisms during the divergence period, subsequently acting as barrier loci upon secondary contact.

The origin of such alleles that started to diverge before the average coalescent time expected from historical reconstructions remains uncertain. Recent studies have emphasized the role of introgression from a distantly related lineage as a source of new adaptations^54–56^, which can possibly play a role in RI^57^. Although we previously found no evidence for contemporary gene flow between *D. labrax* and its closest relative *D. punctatus*^31^, we cannot rule out a possible past admixture with *D. punctatus* or an extinct lineage or the existence of an ancestral population structure^42^. Another possible explanation involves the existence of balanced polymorphisms of various types (e.g., frequency-dependent selection or local adaptation) maintained for a long time in the ancestral population, followed by the fixation of alternative alleles in the derived populations^58^.

Whatever the origin of the alleles that contribute to RI, our results clearly show that barrier loci tend to map preferentially to low-recombining regions. Such pattern may arise through different but non-mutually exclusive mechanisms. First, during isolation, weakly deleterious mutations are more likely to become fixed by drift or due to hitchhiking with a positively selected allele if recombination is reduced^59^. This may subsequently trigger the fixation of compensatory mutations independently in each population, which could become genetic incompatibilities upon contact^60^. Another effect of linked selection is to accelerate lineage sorting^27^, increasing the chance to fix alternative alleles in low-recombining regions during short isolation periods (i.e., <10 *N*_e_ generations). Finally, during secondary contacts, the retention of divergence is facilitated when multiple incompatibility loci combine their effects through linkage, especially if some of these loci are involved in local adaptation^61^. Therefore, the density of selected sites determines, in interaction with recombination, the strength of selection against introgressed tracts and the tendency for increased neutral introgression near chromosome extremities^24,28,30,53,62^. These different effects are likely to be amplified if ancestral variation has been fueled by foreign alleles coming from a distant lineage during the divergence history.

To conclude, our results shed new light on the origin and remolding of genomic islands during the divergence history of European sea bass lineages. Thanks to the use of haplotypic information, we provide a more mechanistic understanding of the complex interplay between linked selection, allele age, and resistance to introgression. The recombination landscape appears to be an essential driver of the observed genomic patterns, influencing both lineage sorting and introgression. Our findings also support that the genomic islands generated by linked selection tend to be disproportionately involved in RI during allopatric speciation, although some of them are purely incidental and are currently being eroded by gene flow. Finally, the probability of introgression in a particular genomic region is negatively related to the level of divergence between alleles, a result possibly indicating that either past admixture or long-term balancing selection has participated to the evolution of reproductive barriers in sea bass.

## Methods

### Whole-genome resequencing and haplotyping

Haplotype-resolved whole genomes were obtained using a phasing-by-transmission approach^63^. Eight parent–offspring trios were generated using 16 wild European sea bass, including 4 males from the Atlantic Ocean (English Channel, ♂_ATL_), 4 males from the eastern Mediterranean sea (2 from Turkey and 2 from Egypt, ♂_E-MED_), and 8 females from the western Mediterranean sea (Gulf of Lion, ♀_W-MED_). This trio design is well adapted to recover haplotype information and use local ancestry for inferring the history of divergence, which does not require large sample sizes. Wild parents were crossed in the laboratory to produce 4 families of ♂_ATL_ × ♀_W-MED_ and 4 families of ♂_E-MED_ × ♀_W-MED_ (Supplementary Fig. 1, Supplementary Table 1). Artificial mating and fish rearing were performed in normal conditions at the Ifremer aquaculture facility under experimental agreement C 34-192-6 and in agreement with the French decree no. 2013 -118 1 February 2013 NOR:AGRG1231951D. For each family, the two parents and one randomly selected descendant were submitted to whole-genome resequencing.

Individual genomes were sequenced to an average depth of 15.5× (Supplementary Table 1, Supplementary Fig. 2) using Illumina paired-end reads of 100 bp. Reference alignment to the sea bass genome^31^ was performed using BWA-mem v0.7.5a^64^. After filtering duplicate reads, the mean coverage depth per family ranged between 11.3× and 17.6×. We followed the Genome Analysis Toolkit (GATK v3.3-0) best practice pipeline^65,66^, applying both base-quality score recalibration and variant-quality score recalibration before calling variants (Supplementary Note 1). Phasing-by-transmission was performed using default parameters. For all downstream analyses, we only used SNPs from parental genomes that were successfully phased using the information contained in the genome of their offspring. SNPs located on the mitochondrial chromosome and the ungrouped fraction of the genome were excluded. Finally, we applied a stringent filter on individual genotype quality (>30) and only retained variants without any missing or excluded genotype. Our final dataset consisted of 2,628,725 SNPs phased into chromosome-wide haplotypes from 14 individuals.

### Detection of introgressed haplotypes

We used Chromopainter v0.04^67^ to perform local ancestry inference and identify migrant tracts resulting from introgression between Atlantic and Mediterranean lineages. The program uses a hidden Markov Model to estimate the probability of Atlantic and Mediterranean ancestry at each variable position along the genome using patterns of haplotype similarity. Each individual was separately considered as a “recipient” and compared to “donor” individuals present in reference Atlantic and Mediterranean populations. A recipient chromosome was reconstructed as a combination of DNA chunks from donor individuals, the donor of each chunk being identified as the most similar haplotype from the reference populations. Therefore, the local ancestry profile of a given recipient chromosome was made of a mosaic of DNA chunks for which the probability to be inherited from either lineages was inferred by Chromopainter.

We developed a method to determine the starting and ending positions of each migrant tract (Supplementary Fig. 3). Atlantic migrant tracts within Mediterranean genomes were delimited by analyzing the probability of Atlantic ancestry inferred by Chromopainter. A given haplotype was considered as truly Atlantic when this probability was >0.95 and as Mediterranean when it was <0.05. Positions with intermediate probabilities lying between 0.05 and 0.95 corresponded to ambiguous regions that were assigned the same ancestry as non-ambiguous neighboring sites. Therefore, an Atlantic tract was defined by a starting position where the inferred probability of Atlantic ancestry reached 0.95 and an ending position where it dropped below 0.05. To avoid bias in the estimated length of introgressed tracts depending on the reading direction of ancestry profiles, we shifted the beginning and ending point of each tract to the closest position with an ancestry probability of 0.5. The presence of Mediterranean migrant tracts within Atlantic genomes were identified in the same way, by analyzing the probability profile of Mediterranean ancestry.

Chromopainter requires non-introgressed reference individuals from every potential source population in order to detect introgressed haplotypes in focal samples. Although the Atlantic lineage is only slightly introgressed by Mediterranean alleles, the Mediterranean lineage is by contrast heavily impacted by gene flow. Therefore, the presence of Atlantic haplotypes in both Atlantic and Mediterranean references populations can confound the accurate prediction of local ancestry by Chromopainter. We thus developed a procedure to reconstruct non-introgressed Mediterranean genomes by removing migrant tracts in a step-wise manner (Supplementary Note 3, Supplementary Fig. 4). Introgressed segments of Atlantic origin were identified by exploiting the different levels of introgression existing between W-MED and E-MED populations (31% in the W-MED compared to 13% in the E-MED population, see Results). This strategy enabled us to reconstruct the ancestral genetic diversity of the Mediterranean populations before secondary gene flow from the Atlantic, which significantly improved the detection of introgressed tracts (Supplementary Note 4, Supplementary Figs 5 and 6).

### Analysis of migrant tract length distribution

The most abundant class of tracts found in low-recombining regions of the sea bass genome have an average length of 50 kb (Supplementary Fig. 7). Using an analytical expectation for the average length of migrant tracts following an admixture pulse^42^, we estimated that these tracts have introgressed the W-MED population approximately 17,000 years BP (Supplementary Note 5). Given that 85% of the Atlantic introgressed tracts found in low-recombining regions are on average >50 kb, the length distribution of migrant tracts is consistent with a post-glacial secondary contact. To assess the goodness-of-fit of the secondary contact model previously inferred from the joint allele frequency spectrum^31^, we compared the length distributions of migrant tracts observed in Atlantic and Mediterranean populations to simulated distributions. We used the coalescent simulator msprime v0.4.0^68^ to generate genome-scale haplotype data with variable recombination rates under the previously inferred secondary contact model^31^ (Supplementary Fig. 8), using the same parameter values. We then analyzed simulated data with Chromopainter to get the genome-wide distribution of migrant tract length in each population and compared it with the observed distributions.

### Testing waves of historical gene flow

The demographic history of Atlantic and Mediterranean sea bass populations was inferred from the length distribution of tracts of IBS using a composite likelihood method^43^. We extended this approach to test for successive waves of gene flow during divergence (Supplementary Note 6). We developed a simple and flexible model that can account for multiple equal-length episodes of divergence and gene flow between two populations (Fig. 3a). Using only nine parameters, the model can represent a large range of demographic scenarios falling into three categories. (i) Continuous migration (an approximation of the Isolation-with-Migration model) is modeled using several contacts (*m* > 1) each containing a single pulse (*n* = 1), with no isolation period separating contact episodes ($T_{{\mathrm{diff}}} \approx 0$). In this scenario, the *m* pulses are therefore continuously distributed along the whole divergence history, with $T_{\mathrm{c}}$ generations separating two consecutive pulses. (ii) Secondary contact with a single period of isolation and gene flow (*m* = 1), including a long enough interruption of gene flow to allow divergence before contact $\left( {T_{{\mathrm{diff}}} > \frac{{T_{\mathrm{C}}}}{n}} \right)$. (iii) Periodic pulses with *m* > 1 and a long enough interruption of gene flow to initiate divergence between two successive contacts ($T_{{\mathrm{diff}}} > 0.1 \times N_{\mathrm e}$ and $T_{{\mathrm{diff}}} > \frac{{T_{\mathrm{C}}}}{n}$). In secondary contact and periodic pulses scenarios, continuous gene flow within contact episodes can be approximated with several admixture pulses sufficiently close in time ($\frac{{T_{\mathrm{C}}}}{n}$ « *N*_e_). Even though every scenario can be modeled using the same number of parameters, the total number of admixture pulses in a given scenario (i.e., the product *m* × *n*) acts as a nuisance parameter. Therefore, we only compared estimated composite likelihoods among scenarios having the same total number of admixture pulses (Fig. 3c and Supplementary Table 2).

The information about the timing of introgression events is expected to be better preserved within low-recombining regions^40,42^. Therefore, we only used sequence information from the low-recombining fraction of the sea bass genome (using *ρ* ≤ 10, the population-scaled recombination rate estimated by ref. ^31^) to infer the demographic divergence history from the length distribution of IBS tracts. Inferences were performed using a grid search on the total number of pulses, making the product *m* × *n* vary from 1 to 10. For every combination of *m* and *n* values, we used 20 independent runs in which we let the 7 other parameters being freely estimated during composite likelihood optimization (Supplementary Table 3).

### Whole-genome alignment with an outgroup species

We used the 35,012 scaffolds from the *Morone saxatilis* genome assembly (www.ncbi.nlm.nih.gov/assembly/GCA_001663605.1/; sequence length = 585.2 Mb; N50 = 30 kb) to perform alignments against the reference genome of *D. labrax*. The Mauve Contig Mover tool^69^ from the Mauve software v2.4.0^70^ was used to match every scaffold from *M. saxatilis* to the *D. labrax* genome and generate a list of scaffolds matching to each *D. labrax* chromosome. Matching scaffolds were properly ordered and assembled along each chromosome to generate 24 pseudo-chromosomes using the software Abacas version 1.3.1^71^. We then aligned *M. saxatilis* pseudo-chromosomes to the *D. labrax* genome using the algorithm progressiveMauve in order to identify insertions in the *M. saxatilis* genome that were removed to only conserve homologous sites present in *D. labrax*. After whole-genome alignment, mapped scaffolds were evenly distributed among the 24 sea bass chromosomes, providing outgroup information for 52% of the *D. labrax* genome. The genome-wide average nucleotide divergence between *D. labrax* and *M. saxatilis* was 4.35% (s.d. = 2.36%).

### Population genomics statistics

We used several complementary approaches to assess the level of divergence and introgression between lineages. First, the extent of genetic differentiation between Atlantic and Mediterranean populations of *D. labrax* was evaluated using both relative (*F*_ST_^72^) and absolute (*d*_XY_^73^) measures of divergence. We used Vcftools v0.1.11^74^ and MVFTools v3.0^75^ to calculate the average *F*_ST_ and *d*_XY_ in non-overlapping 100 kb windows. Second, we used Chromopainter outputs to directly measure the percentage of positions occupied by migrant tracts and calculate their average length in 100 kb windows. Finally, we calculated the minimum and maximum pairwise distance between haplotypes sampled from Atlantic and Mediterranean populations (*d*_XYmin_ and *d*_XYmax_) and divided these values by the divergence between *D. labrax* and *M. saxatilis* (*d*_out_) in each window to compute RND_min_^76^ and RND_max_ statistics. The RND_min_ ratio is more sensitive to low-frequency migrant tracts than *F*_ST_ and *d*_XY_. When introgression occurs at a genome-wide scale, the highest RND_min_ values indicate regions of reduced introgression, whereas RND_max_ rather reflects the maximal absolute divergence among haplotypes irrespective to introgression. Both statistics are robust to mutation rate variation across the genome.

### Testing for RI

In order to test whether genomic islands are actively reshaped by migration and selection, we used simulations to generate predictions under the secondary contact scenario assuming different selective effects. More specifically, we evaluated the influence of BGS and RI on the distribution of *F*_ST_, *d*_XY_, and RND_min_ statistics compared to neutral expectations. The effect of RI and BGS in low-recombining regions were approximated by respectively reducing the effective population size (*N*_e_)^77^ and the effective migration rate (*m*_e_)^28^ compared to neutral loci. We used msprime v.0.0.4^68^ to simulate a post-glacial secondary contact model under three different selection scenarios. (i) A neutral model parameterized using the values inferred from the joint allele–frequency spectrum^31^ (i.e., similar to Fig. 2). (ii) A BGS model with an increased rate of lineage sorting and neutral introgression. Three possible levels of BGS were applied to both ancestral and derived populations (i.e., 0.5 × *N*_e_, 0.2 × *N*_e_, and 0.1 × *N*_e_). The middle range value was empirically estimated to correspond to the reduction in nucleotide diversity observed in low compared to highly recombining regions due to linked selection^31^. Finally, (iii) A BGS+RI model with an increased rate of lineage sorting and a decreased rate of introgression. The effective population size was reduced to 0.2 × *N*_e_ in both ancestral and derived populations, and the effective migration rate was reduced to ~0.2 × *m* compared to neutral regions, as previously inferred for genomic island loci^31^. For each model, we simulated a 150 Mb chromosome with an average recombination rate corresponding to the mean value calculated across the sea bass genome (6.85 cM/Mb). Divergence and introgression statistics were calculated in non-overlapping 100 kb windows using Vcftools^74^ for *F*_ST_ and MVFtools^75^ for *d*_XY_ and RND_min_. To test which simulated model best explains our data, we compared the joint distributions of *d*_XY_, *F*_ST_, and RND_min_ obtained by simulations under each model to the data. For each 100 kb window, we calculated the mean Euclidean distance between the *Z*-scored values of observed and simulated statistics under each of the three models (i.e., Neutral, BGS, and BGS+RI). Models were then ranked by their Euclidean distance to observed data, to determine the best model for each 100 kb window.

### Data availability

Sequence reads have been deposited in the GenBank Sequence Read Archive under the accession code BioProject PRJNA472842.

## Electronic supplementary material

Supplementary Information
