# Publisher Correction: The origin and remolding of genomic islands of differentiation in the European sea bass

**DOI:** 10.1038/s41467-018-05359-2

**Published:** 2018-07-27

**Authors:** Maud Duranton, François Allal, Christelle Fraïsse, Nicolas Bierne, François Bonhomme, Pierre-Alexandre Gagnaire

**Affiliations:** 1 Institut des Sciences de l’Evolution de Montpellier - UMR5554 UM-CNRS-IRD-EPHE, Place Eugène Bataillon, 34095 Montpellier, France; 2 Université de Montpellier, Place Eugène Bataillon, 34095 Montpellier, France; 3 MARBEC, Université de Montpellier, Ifremer-CNRS-IRD-UM, 34250 Palavas-les-Flots, France

Correction to: *Nature Communications* 10.1038/s41467-018-04963-6; published online 28 June 2018.

The originally published version of this Article contained errors in Fig. 5, whereby the sign for Spearman’s rho was incorrect in panels b and c. These errors have now been corrected in both the PDF and HTML versions of the Article.

