# Supplementary material for: The origin and remolding of genomic islands of differentiation in the European sea bass
Source: Nat Commun. 2018 Jun 28;9:2518. doi: 10.1038/s41467-018-04963-6 (PMC6023918; doi:10.1038/s41467-018-04963-6)
Supplement: Supplementary file 1 — Supplementary Information [file 41467_2018_4963_MOESM1_ESM.pdf]

Supplementary Information

Duranton et al.

The origin and remolding of genomic islands of differentiation in the European sea bass

Supplementary Note 1: Whole genome resequencing and haplotyping......................... 2

Supplementary Note 2: Analysis of spatial population structure.................................. 5

Supplementary Note 3: Detection of introgressed haplotypes and reconstruction of ancestral Mediterranean genomes 5

Supplementary Note 4: Effectiveness of ancestral reconstruction................................. 8

Supplementary Note 5: Analysis of migrant tract length distribution 10

Supplementary Note 6: Testing waves of historical gene flow.....................................12

Supplementary References 21

Supplementary Note 1: Whole genome resequencing and haplotyping

Haplotype-resolved whole genomes were obtained using a phasing-by-transmission approach. Experimental crosses were produced between wild sea bass to generate parent-offspring trios and phase parental genomes using their offspring (Supplementary Fig. 1). All experimental procedures were conducted at Ifremer's experimental aquaculture infrastructure (agreement for experiments with animals: C 34-192-6) in agreement with the French Decree n° 2013 –118 1st February 2013 NOR: AGRG1231951D (which transposes Directive 2010-63-EU into research practice for the Care and Use of Laboratory Animals). No specific agreement was required according to Directive 2010-63-EU, article 1.5 (i.e. practices not likely to cause pain, suffering, distress or lasting harm equivalent to, or higher than, that caused by the introduction of a needle in accordance with good veterinary practice are excluded from the Directive), since the fish were reared in normal conditions.

Whole genome sequencing libraries were prepared separately for each of the 24 individuals using the SPRIworks Library Preparation System (Beckman Coulter) to select 350-450bp DNA fragments. The three individual libraries from each trio were pooled and sequenced on a separate lane of an Illumina Hi-Seq 2500 platform using 2×100pb PE reads at the LIGAN-PM Genomics platform (Lille, France) (Supplementary Table 1). Variant discovery and haplotype calling was performed following the GATK version 3.3-0-g37228af best practice pipeline. Raw reads were first aligned to the sea bass reference genome[1] using BWA-mem version 0.7.5a[2] and duplicates were marked using Picard version 1.112 (http://broadinstitute.github.io/picard/index.html). The following steps were performed using GATK, starting with local realignment around indels, individual variants calling using the HaplotypeCaller, joint genotyping, genotype refinement using family priors and hard-filtering of variants to retain the most confident SNPs and indels (Filter Expression: QD<10; MQ<50; FS>7; MQRankSum<-1.5; ReadPosRankSum<-1.5). This subset of high-quality variants was then used to recalibrate base quality scores using the BQSR algorithm. The HaplotypeCaller was ran again on recalibrated sequence data to call individual variants, followed by joint genotyping, variant quality score recalibration using the VQSR algorithm with the previously identified subset of high-quality variants, and genotype refinement using family-based priors. Variants were then filtered to exclude low-quality genotypes with a GQ score <30. All trios were finally phased given parents and child genotype likelihoods and a mutation prior of 10^{-8} for *de novo* mutations using the PhaseByTransmission algorithm with default parameters. Only sites where parent/child transmission could be determined unambiguously were phased (excluding Mendelian violations and uninformative sites). In addition to phasing based on transmission information within trios, we also scored physical phasing information with the HaplotypeCaller algorithm when available (*i.e.* for closely linked variable sites located on the same read pair). For all downstream analyses, we only used parental genomes and only retained SNPs that were successfully phased using the information contained in their children's genome. Two female genomes were excluded prior to using family-based priors in the GATK pipeline since they appeared to be misidentified mothers in their trios

(T3F and T4F, Supplementary Table 1), which were therefore treated as father-child duos for phasing. Finally, we used VCFtools version 0.1.11[3] to only retain SNP variants (indels were removed) without missing genotype in the 14 parental genomes. Variants located on the ungrouped fraction of the genome (i.e. non-assembled scaffolds) as well as on the mitochondrial chromosome were excluded. Our final dataset consisted of 2,628,725 SNPs phased into chromosome-wide haplotypes from 4 males of Atlantic origin, 6 females from the western Mediterranean Sea, and 4 males from the eastern Mediterranean Sea. Mapping summary statistics and depth of coverage for each individual included in this study are detailed in Supplementary Table 1 and Supplementary Fig. 2.

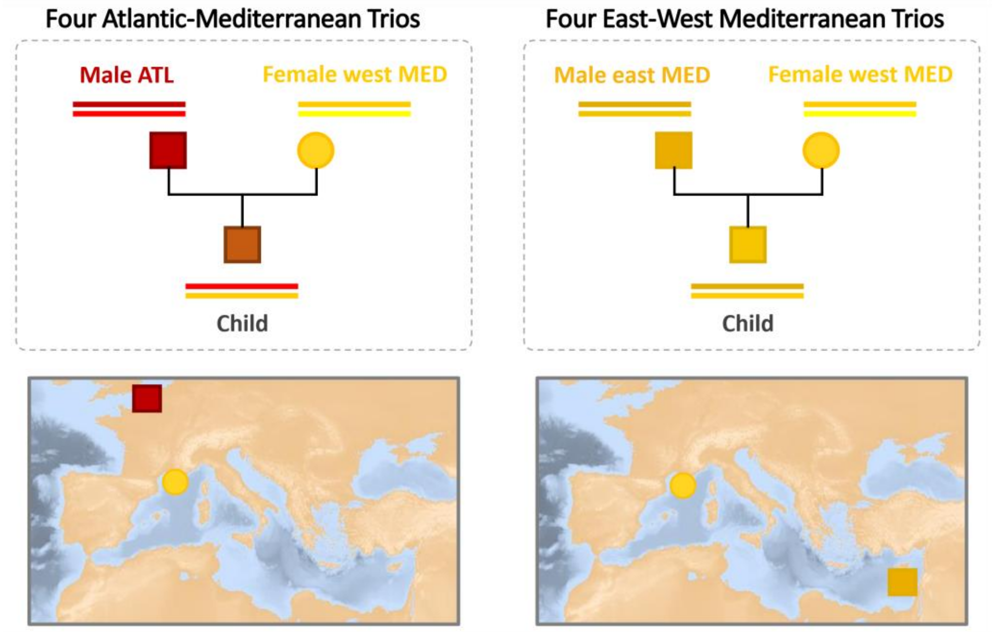

Supplementary Figure 1 – **Schematic representation of the two different types of crossings generated to produce sea bass trios**. Four trios were obtained by crossing parents from Atlantic and Mediterranean sea bass lineages (left panel), and four trios by crossing parents from eastern and western Mediterranean populations (right panel). The geographic area of parents' origin is indicated for each type of crossing. Male parents are represented by the squares and females by circles. The red color symbolizes the Atlantic lineage, and the two Mediterranean populations are colored in light yellow (western Mediterranean) and dark yellow (eastern Mediterranean).

Supplementary Table 1 – **Summary statistics of sequencing and mapping data for each individual.**

Individual	Unpaired reads examined	Reads pairs examined	Unmapped reads	Unpaired read duplicates	Read pair duplicates	Percent duplication	Estimated library size
T1C	1081927	66809192	2616613	247413	884126	0.014964	2540848981
T1F	2668171	47343728	4479891	1249028	5158971	0.118812	201992483
T1M	1139190	61374922	2718960	166362	730774	0.01314	2611630891
T2C	3018619	75084510	5055081	940730	6420707	0.089969	414227864
T2F	1468390	84761271	3688068	352752	2419315	0.030361	1466128093
T2M	6397183	13596684	7005255	1458522	1688617	0.143962	50139501
T3C	802017	43185540	1938499	138166	378074	0.010259	2502021318
T3F	1369036	55513323	3683430	381458	1792017	0.035282	848979317
T3M	1097071	57112388	3451939	241435	758592	0.01525	2177220972
T4C	1338977	45811338	2851255	202624	921213	0.021999	1147167323
T4F	2198974	66545468	7145418	984486	2123317	0.038666	1036654039
T4M	1339876	51424532	2990880	197138	1253063	0.025946	1051212241
T5C	1338511	59270268	3003509	310932	1460799	0.026965	1197082582
T5F	1980060	45252463	3513888	528965	7825578	0.174949	115496763
T5M	1062988	56612039	2660466	168302	1131827	0.021279	1415430537
T6C	2578026	63399201	4439602	717966	6377110	0.104132	294754616
T6F	1931413	51031022	3829965	389923	1962146	0.041485	659999628
T6M	3583979	7260050	3926337	1129388	1351914	0.211732	17009615
T7C	758612	60554046	2344194	234454	927538	0.017146	1982688539
T7F	1725368	47251888	3494428	303753	2317839	0.05133	471050437
T7M	814605	53756361	2280031	119211	744762	0.014851	1966354587
T8C	1398328	63282016	4178788	395006	1133963	0.02081	1775204766
T8F	1272884	64616082	4206008	464481	2339433	0.039411	877042879
T8M	1064127	53646079	3725473	245293	670915	0.014647	2180868161

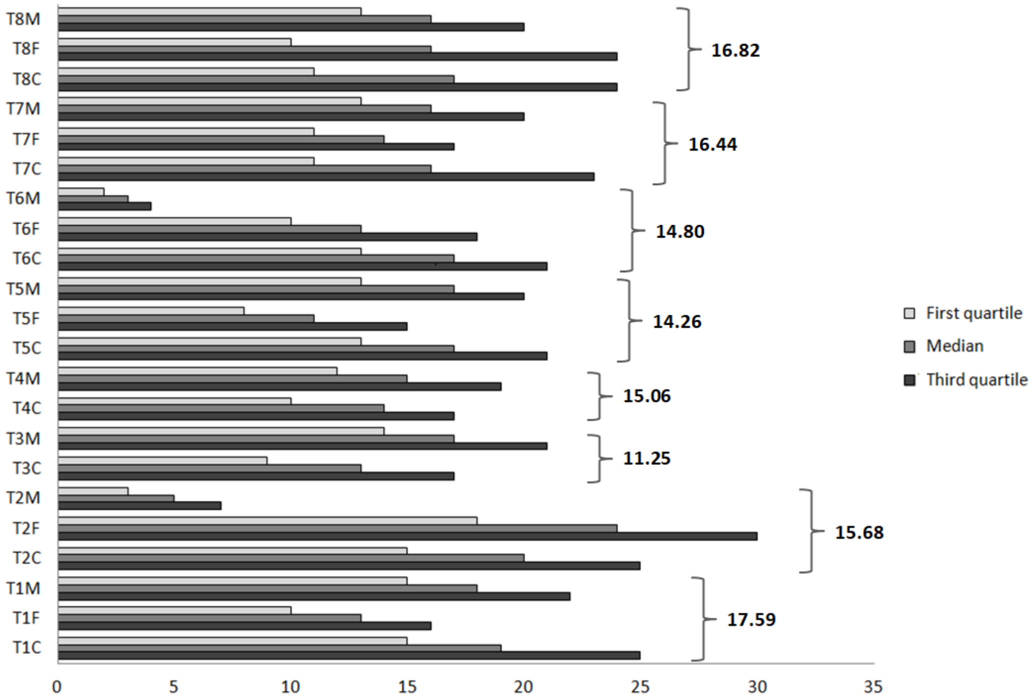

Supplementary Figure 2 – **Depth of coverage per individual.** Median (dark gray), first (light gray) and third (black) quartile of the depth of coverage for the 4 Atlantic males (T1M – T4M), the 4 eastern Mediterranean males (T5M – T8M), the 6 western Mediterranean females (T1F – T8F), and the descendant of each family (T1C – T8C). The average coverage depth of each family is indicated on the right side of the bars.

Supplementary Note 2: Analysis of spatial population structure

In order to determine the genetic position of the newly sequenced genomes with respect to the range-wide population structure of the European sea bass, we merged the new SNP dataset containing 2,628,725 SNPs with a RAD-derived SNP dataset containing 134,815 SNPs (mean read depth per SNP ranging from 10X to 150X, minor allele frequency (MAF) threshold = 0.01, genotyping rate threshold = 0.8). The RAD SNP dataset consists of 112 individuals from six locations: two in the Atlantic (Biarritz (France), N = 12; Mondego Estuary (Portugal), N = 26), two in the western Mediterranean (Palavas/Mauguio lagoon (France), N = 40, Annaba/Mellah lagoon (Algeria), N = 25) and two in the eastern Mediterranean (Syracuse (Italy), N = 1, Zarzis/El Biben lagoon (Tunisia), N = 8). Some individuals were already analyzed[1] and other were specifically added for this study. RAD SNP genotypes were obtained using the same reference mapping/genotyping pipeline as the one used for the whole genomes, but without using family priors. The two VCFs files derived from the whole genomes and the RAD-Sequencing data were merged together using VCFtools[3].

We then used the R package adegenet[4] to perform a Principal Component Analysis (PCA) of the combined dataset using only high-quality common variants. A total of 13,094 SNPs with a minor allele frequency > 0.10 and a genotyping rate greater than 0.9 were used for this analysis.

Supplementary Note 3: Detection of introgressed haplotypes and reconstruction of ancestral Mediterranean genomes

We used Chromopainter[5] with 150 iterations for the EM algorithm (expectation-maximization) to estimate the probability for each position along each haplotype to come from the Atlantic and Mediterranean population. Given that Chromopainter requires an estimate of the local recombination rate, we used the population recombination rate ($\rho = 4N_e r$) averaged between Atlantic and Mediterranean populations, already estimated in a previous study[1]. We then developed a method to analyze the ancestry probability profiles produced by Chromopainter to identify the beginning and the end of each introgressed tract (Supplementary Fig. 3).

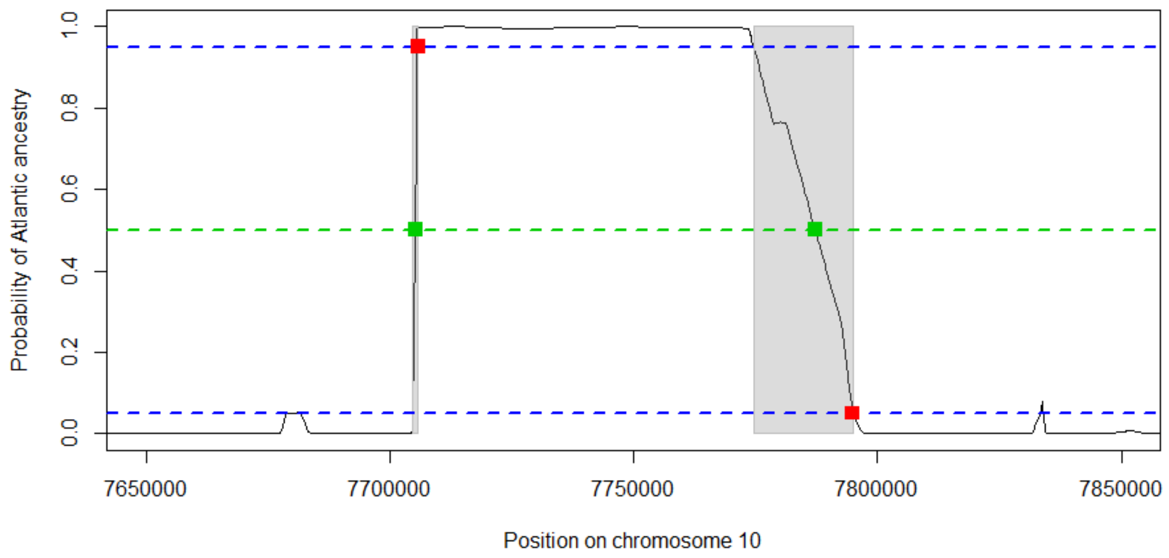

Supplementary Figure 3 – **Probability profile of Atlantic ancestry for a Mediterranean sample haplotype along a 200kb region located on chromosome 10.** The probability of Atlantic ancestry was determined at each SNP by Chromopainter taking the local recombination rate into account. The squares represent the starting and ending positions of an introgressed tract of Atlantic origin before (red) and after (green) shifting the positions to the nearest point with a 0.5 probability (green dotted line) in the zone of uncertainty (grey rectangles). The blue dotted lines represent the probability thresholds used to consider a haplotype as truly Atlantic (0.95) or truly Mediterranean (0.05).

We reconstructed a non-introgressed Mediterranean population by removing introgressed Atlantic tracts to generate reference samples to be used in Chromopainter. We identified and removed introgressed Atlantic tracts within Mediterranean genomes using a three-step procedure (Supplementary Fig. 4). First, we used the Atlantic and E-MED populations as references to identify Atlantic haplotypes introgressed in the W-MED population, and reciprocally for the eastern Mediterranean population. We then removed the identified Atlantic tracts and replaced each of them by tracts identified as purely Mediterranean. The Mediterranean haplotypes used for replacement were obtained by determining the majority consensus sequence from all the sequences identified as purely Mediterranean at these positions. One consensus was generated for each Mediterranean population separately. Therefore, the gaps created by the removal of introgressed Atlantic tracts were filled with the most frequent allele found locally in the Mediterranean population considered. This step was repeated a second time using the Mediterranean populations reconstructed without introgressed tracts in the previous step as new references. This additional step aimed at removing residual tracts of Atlantic ancestry that could not be detected in the first step due to high rates of introgression in some regions of the genome in both eastern and western Mediterranean populations. After this step, only genomic regions that are completely swamped by Atlantic alleles should remain undetected, which is probably very rare. The two resulting Mediterranean populations obtained after these two steps should therefore correspond to the Mediterranean ancestral population before the beginning of gene flow. They were used in a third and last step as Mediterranean reference populations to identify migrant tracts (Supplementary Fig. 4).

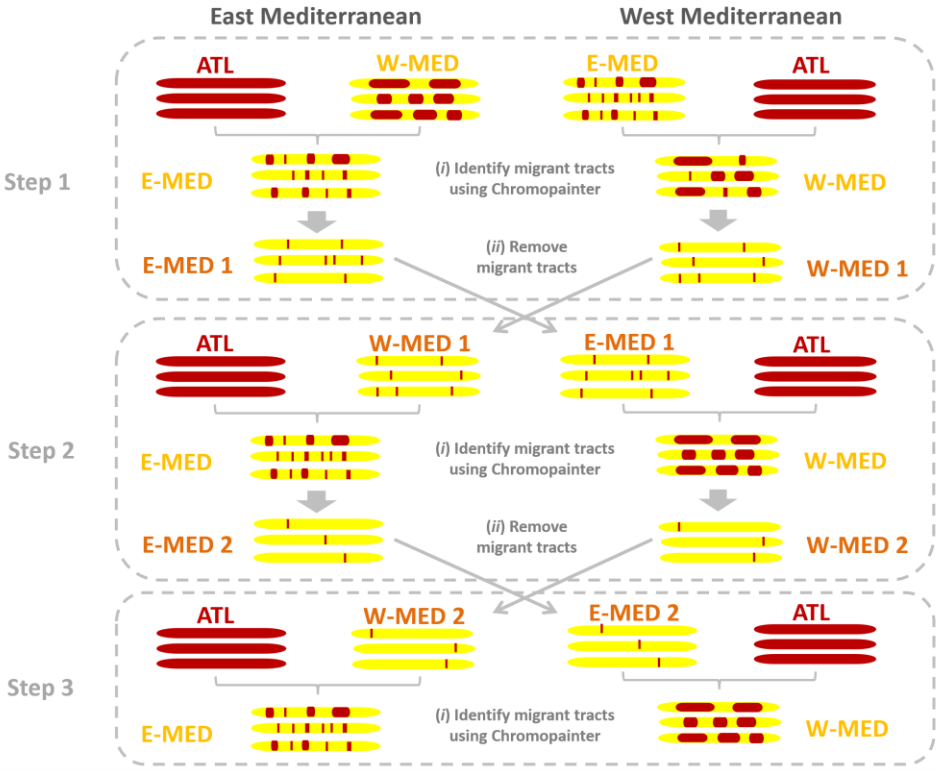

Supplementary Figure 4 – **Schematic overview of the three steps used to reconstruct the ancestral diversity of Mediterranean genomes and identify migrant tracts**. For each step, 3 chromosomes are represented per population for illustration. The red color is used for Atlantic DNA fragments and the yellow color for Mediterranean DNA fragments.

Supplementary Note 4: Effectiveness of ancestral reconstruction

We evaluated the effectiveness of our strategy to reconstruct ancestral Mediterranean genomes. Genome-wide differentiation measures showed that the E-MED population is genetically more differentiated from the Atlantic population than the W-MED population (Supplementary Fig. 5A). This is particularly true for genomic regions with intermediate F_{ST} values. If this difference is due to gene flow, then the W-MED population should be more strongly impacted by introgression than the E-MED population. Indeed, we found that the difference in F_{ST} measured between Atlantic and W-MED versus Atlantic and E-MED (*i.e.* $F_{ST\ (E\text{-}MED/ATL)}$ - $F_{ST\ (W\text{-}MED/ATL)}$) was positively correlated with the differential of introgression (*i.e.* $F_{Introgression\ (W\text{-}MED)}$ - $F_{Introgression\ (E\text{-}MED)}$) between W-MED and E-MED (Supplementary Fig. 5B). This result confirms that the genetic differences observed between the two Mediterranean populations are explained by an increased frequency of introgression within western compared to eastern Mediterranean genomes. As a corollary, we also show that our strategy to reconstruct ancestral Mediterranean genomes generates very similar results between W-MED and E-MED populations. Indeed, genetic differentiation between Atlantic and ancestral W-MED is highly positively correlated to genetic differentiation between Atlantic and ancestral E-MED, with a regression slope close to 1 confirming that reconstructed ancestral Mediterranean populations are equally differentiated from the Atlantic population (Supplementary Fig. 5C). This suggests that the method to reconstruct the ancestral state of Mediterranean genomes independently between W-MED and E-MED has efficiently reconstructed the same gene pool, supposed to reflect the genetic composition of the Mediterranean population before the beginning of gene flow. We note that this approach only treats the effect of introgression and does not address the coalescence of Mediterranean alleles to reconstruct ancestral genomes. It may also bias the frequency of Mediterranean alleles by replacing Atlantic tracts by the most frequent Mediterranean haplotype. However, this does not affect our approach since we mostly used the reconstructed ancestral genomes as references to improve the detection of introgressed tracts.

Using the reconstructed Mediterranean ancestral populations as references in Chromopainter improved the detection of migrant tracts within Mediterranean genomes in two different ways. First, the use of reconstructed reference populations allowed the detection of previously undetected Atlantic haplotypes (Supplementary Fig. 6A). Second, it also increased the precision of tract length estimation (Supplementary Fig. 6B). In most cases, the removal of Atlantic tracts in the Mediterranean reference populations increased the Atlantic ancestry probability in the genomic regions where introgression was strong, thus enabling a better detection and more precise measurement of migrant tracts. Finally, estimated ancestry probability profiles were very similar using the ancestral Mediterranean population reconstructed after one or two steps of introgressed tracts removal (Supplementary Fig. 6). This suggests that two steps were enough to efficiently detect and remove Atlantic tracts within Mediterranean genomes, and that adding a third step would not significantly improve the approach.

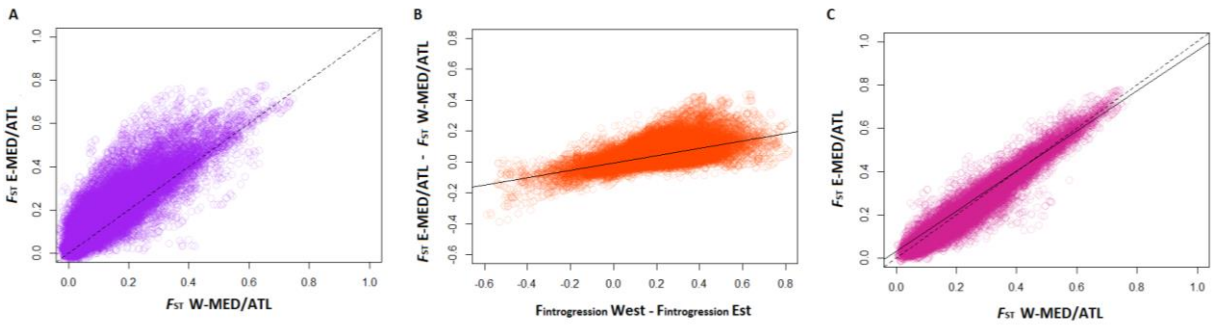

Supplementary Figure 5 – **Genome-wide correlations between different statistics averaged in 100 kb windows.** A. Correlation of genetic differentiation (F_{ST}) measured between Atlantic and western Mediterranean populations (F_{ST} W-MED/ATL) and between Atlantic and eastern Mediterranean populations (F_{ST} E-MED/ATL). The dotted line represents the equation y = x. B. Correlation between the difference in F_{ST} between the comparisons E-MED/ATL and W-MED/ATL and the differential of introgression between western and eastern Mediterranean populations. The black line is the linear regression based on the data. C. Correlation of genetic differentiation (F_{ST}) measured between Atlantic and ancestral western Mediterranean (F_{ST} W-MED/ATL) and between Atlantic and ancestral eastern Mediterranean (F_{ST} E-MED/ATL). The dotted line represents the equation y = x and the black line shows the linear regression based on the data.

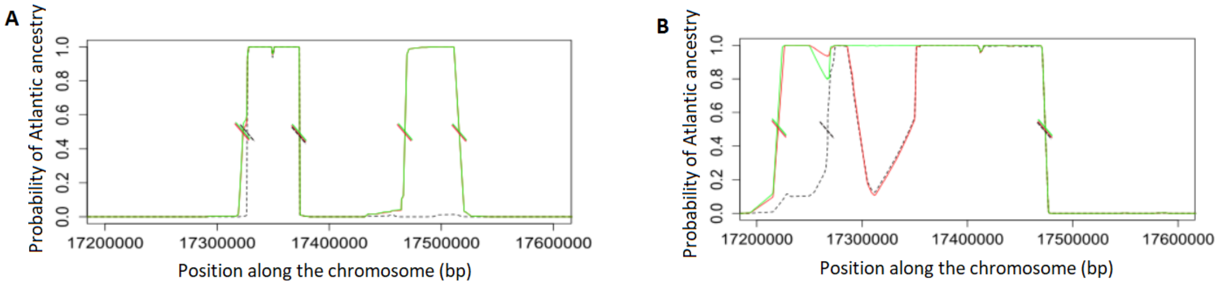

Supplementary Figure 6 – **Examples of Atlantic ancestry probability profiles inferred along chromosome 1A for one chromosome haplotype of a western Mediterranean individual**. Estimation of local ancestry was performed using the Atlantic population as reference together with either the contemporary E-MED population (black dotted lines), the reconstructed ancestral E-MED population after one (red lines) or two rounds of introgressed tracts removal (green lines). Oblique marks indicate the starting and ending positions of detected Atlantic tracts using each of the three different Mediterranean populations as reference (black, red, green). Using a reconstructed ancestral Mediterranean population allows to A. detect new migrant tracts and B. improve the estimation of migrant tract length.

Supplementary Note 5: Analysis of migrant tract length distribution

The distribution of migrant tract length is informative about the timing and intensity of the genetic exchanges that have occurred between populations. After introgression into a recipient population, a migrant tract is progressively eroded over generations due to recombination with the introgressed genetic background[6,7]. The length of a migrant tract thus depends on the number of generations elapsed since introgression (t) and on the local recombination rate of the genomic region in which it is located (r). The average length of migrant tracts ($\overline{L}$) following a single pulse of admixture occurring t generations ago can be approximated by $\overline{L} = [\ 1 - f\ \ r\ \ t - 1\ ]^{-1}$, where f is the fraction of the population replaced by migrants[8]. We used the previous equation to estimate the date of introgression of the most abundant class of tracts found in low-recombining regions of the sea bass genome, which have an average length of 50 kb (Supplementary Fig. 7). Using $f = 0.31$ (the mean frequency of introgression estimated in W-MED individuals, see results), $r = 8.45{\times}10^{-9}$ M/pb (estimated in a previous study[1]) and assuming a generation time of 5 years[1], we calculated that these tracts have introgressed approximately 17,000 years BP. Since 85% of the introgressed tracts of Atlantic origin found in low-recombining regions are on average longer than 50kb, they probably introgressed the W-MED population less than 17,000 years ago. Reciprocally, only 15% of the tracts are on average shorter than 50 kb and have therefore possibly introgressed the W-MED population during an older contact episode. Therefore, the secondary contact model previously inferred without using linkage information[1] appears consistent with this result. In order to assess its goodness-of-fit more thoroughly, we compared the observed length distributions of migrant tracts with the ones obtained by simulation under the secondary contact model.

Coalescent simulations under the secondary contact model included a third, non-introgressed Mediterranean population, to use later as a reference in Chromopainter. To do so, we splitted the Mediterranean population into two replicates at the beginning of the secondary contact so that only one replicate is affected by gene flow from the Atlantic (Supplementary Fig. 8). Model parameter values corresponded to the ones inferred previously[1]. However, since this model included two different categories of loci experiencing different effective migration rates, we used the weighted average migration rate taking into account the relative fraction of the genome occupied by each category of loci. A simulation was performed independently for each chromosome using the local recombination rate previously inferred for each population[1]. Finally, we used Chromopainter to get the length distribution of migrant tracts for each of the two simulated populations.

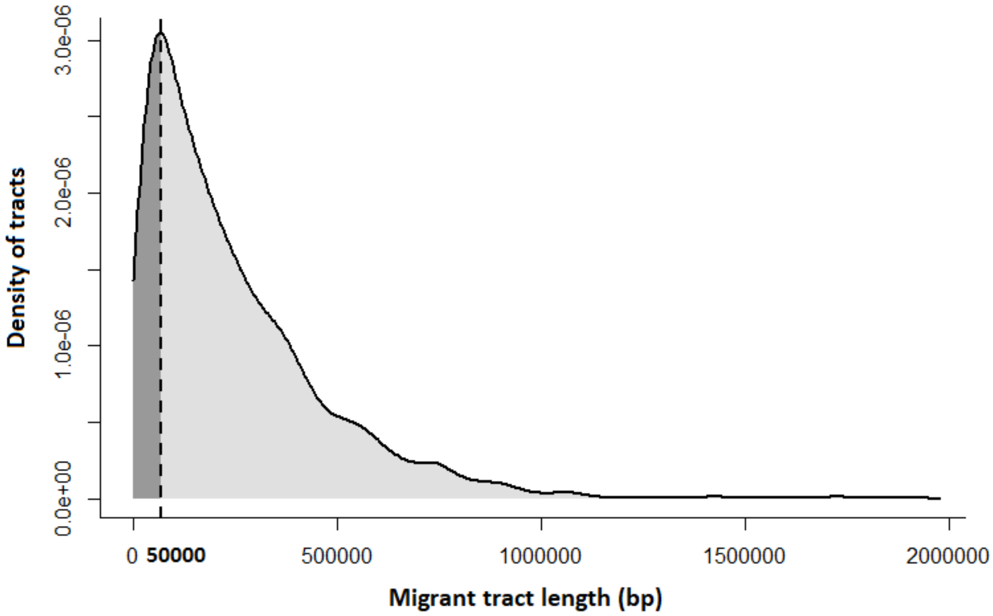

Supplementary Figure 7 – **Distribution of the average length of Atlantic migrant tracts found in 100 kb windows located in low-recombining regions of W-MED genomes.** The vertical dotted line shows the most abundant class length represented by windows with an average tract length of 50 kb. About 15 % of the windows have a lower mean tract length (dark grey area) and 85% of the windows a higher mean tract length (light grey area). Regions are considered as low-recombining if $\rho \leq 10$, with ρ being the population-scaled recombination parameter previously estimated[4].

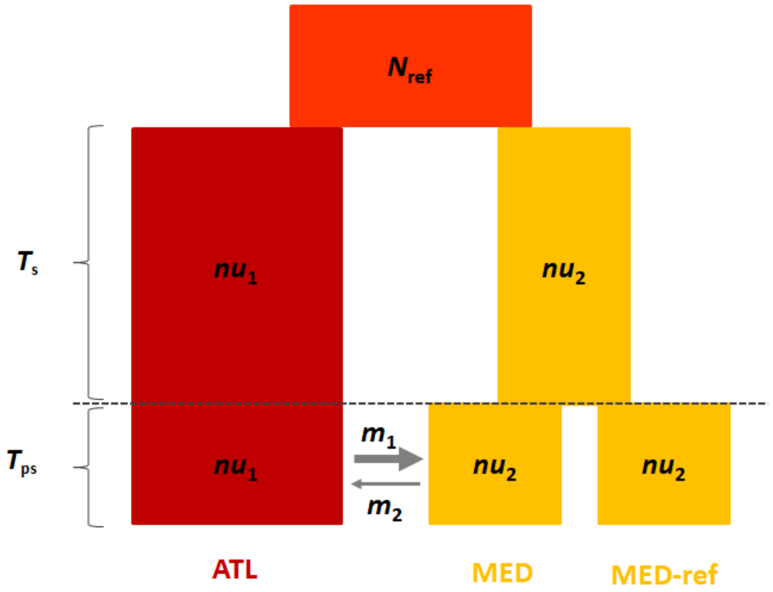

Supplementary Figure 8 – **Schematic representation of the model implemented to simulate the migrant tract length distribution**. An ancestral population of size N_{ref} splits into two derived populations of size nu_1 and nu_2, which evolve without exchanging genes during T_s generations. At the end of this allopatric divergence period, the Mediterranean population splits into two populations of size nu_2, and only one of them exchanges genes with the Atlantic population during T_{ps} generations. Migration from ATL to MED occurs at rate m_1, and at rate m_2 in the opposite direction. MED-ref was used as a reference population together with the Atlantic population for the detection of introgressed tracts in Chromopainter to detect introgressed tracts.

Supplementary Note 6: Testing waves of historical gene flow

We tested whether there have been several periods of allopatric isolation and secondary contact between the Atlantic and Mediterranean Sea bass lineages. To do so, we used a method[9] that exploits the information contained in a collection of pairwise sequence alignments by summarizing the length distribution of tracts of identity-by-state (IBS). An L-base IBS tract is defined as a segment of L contiguous identical base pairs between two consecutive SNPs. The distribution of IBS tracts shared between DNA sequences from different populations contains information about population divergence, paste gene flow and genetic diversity that existed at different time in the populations. It can therefore be used to jointly estimate the timing and magnitude of past admixture events, population divergence times and changes in effective population size. The method uses an approximate formula to predict the expected IBS tract length distribution within and between populations under different models of historical divergence with or without gene flow. The empirical and predicted distributions are then compared to maximize a composite likelihood function.

We developed a flexible model that accounts for a large diversity of demographic histories using nine parameters (Supplementary Fig. 3B). The model can represent three different categories of scenarios, (*i*) continuous migration, (*ii*) secondary contact and (*iii*) periodic pulses. The three categories of scenarios were then compared to each other for each value of $m \times n$ to identify the best model. Note that the periodic pulses scenario is only defined for $m \times n \in \{4, 6, 8, 9, 10\}$ (Supplementary Fig. 3C). Parameter bounds were set as follows: [0.1; 2] for the effective population sizes, [0.001; 0.5] for the admixture fractions, [0.001; 1] for the time parameters, except for the periodic pulses scenario in which the bounds of T_{diff} were set to [0.1; 1] to force divergence periods to be at least $0.1N_e$ generations long. The genetic diversity θ ($4N_e\mu$) and the population scaled recombination rate ρ ($4N_e r$) parameter were estimated from previous data[1], and set to $\theta = 0.001$ and $\rho = 0.001$ with $N_e = N_{ref} = 100{,}000$. Finally, the binning scheme was adjusted to better capture the signature of recent admixture, using 32 bins with b_0=100 and b_{i+1}=1.3×b_i. Therefore, IBS tracts shorter than 100 base pairs were not considered for likelihood optimization, but no upper threshold was set on the longest IBS tracts.

Supplementary Table 2 – **Results of model fitting for 27 combinations of *m* and *n* parameters.** The log(*Likelihood*) value is showed for the best run obtained over 20 optimizations for each (*m*:*n*) combination.

Number of pulses	1	2	3	4	5	6	7	8	9	10
Secondary contact	(1:1) -245443	(1:2) -126504	(1:3) -85028	(1:4) -73381	(1:5) -66147	(1:6) -61448	(1:7) -55760	(1:8) -55824	(1:9) -54024	(1:10) -52613
Continuous migration		(2:1) -133220	(3:1) -90439	(4:1) -76903	(5:1) -70269	(6:1) -65633	(7:1) -62301	(8:1) -59814	(9:1) -57892	(10:1) -56366
Periodic pulses				(2:2) -75199		(2:3) -66411 (3:2) -67636		(2:4) -61908 (4:2) -66536	(3:3) -64122	(2:5) -59102 (5:2) -67111
Best model	SC	SC	SC	SC	SC	SC	SC	SC	SC	SC

Supplementary Table 3 – **Results of model fitting for 27 combinations of *m* and *n* parameters.** Details of parameter values are provided for the best run obtained over 20 optimizations for each *m*:*n* combination. The best-fit model (1:10) is highlighted in red.

m	*n*	N_1	N_2	N	T_c	T_{diff}	f_1	f_2	*Likelihood*	Model
1	1	0.1408	0.3534	0.8956	0.242	0.001	0.5	0.0445	-245443	Sec. contact
1	2	0.119	0.4057	1.0668	0.158	0.2538	0.5	0.0372	-126504	Sec. contact
1	3	0.109	0.469	1.1813	0.2402	0.2057	0.5	0.001	-85028	Sec. contact
1	4	0.1621	0.429	1.2345	0.3189	0.1923	0.4118	0.032	-73381	Sec. contact
1	5	0.1883	0.4135	1.2682	0.3617	0.1837	0.3573	0.0403	-66147	Sec. contact
1	6	0.204	0.4061	1.2903	0.391	0.1746	0.3191	0.0421	-61448	Sec. contact
1	7	0.221	0.4024	1.3191	0.4824	0.1218	0.3038	0.0467	-55760	Sec. contact
1	8	0.2223	0.3988	1.3163	0.4301	0.1572	0.2659	0.0406	-55824	Sec. contact
1	9	0.2286	0.3967	1.3244	0.442	0.1492	0.2463	0.0392	-54024	Sec. contact
1	10	0.2332	0.3951	1.3306	0.4561	0.1417	0.2296	0.0377	-52613	Sec. contact
2	1	0.1315	0.3862	1.0426	0.1887	0.001	0.5	0.0468	-133220	Continuous
2	2	0.1284	0.4493	1.2087	0.1312	0.1	0.4645	0.0142	-75199	Periodic
2	3	0.1899	0.4081	1.295	0.1791	0.1	0.3454	0.0437	-66411	Periodic
2	4	0.2119	0.3993	1.3476	0.2061	0.1	0.2873	0.0448	-61908	Periodic
2	5	0.2247	0.3956	1.3769	0.2241	0.1	0.248	0.0425	-59102	Periodic
3	1	0.1107	0.4432	1.1433	0.1399	0.001	0.5	0.0104	-90439	Continuous
3	2	0.1898	0.4052	1.3222	0.0951	0.1	0.3501	0.0482	-67636	Periodic
3	3	0.2157	0.396	1.3854	0.1217	0.1	0.27	0.0465	-64122	Periodic
4	1	0.1166	0.4662	1.1956	0.1096	0.001	0.4677	0.001	-76903	Continuous
4	2	0.2075	0.3974	1.3831	0.0655	0.1	0.2943	0.0495	-66536	Periodic
5	1	0.1625	0.4264	1.2291	0.0973	0.001	0.3883	0.0293	-70269	Periodic
5	2	0.2109	0.3965	1.3981	0.0382	0.1	0.2502	0.044	-67111	Periodic
6	1	0.1825	0.4141	1.2514	0.0846	0.001	0.3447	0.0357	-65633	Continuous
7	1	0.1949	0.4078	1.2677	0.0744	0.001	0.3123	0.0375	-62301	Continuous
8	1	0.2044	0.4033	1.2807	0.0664	0.001	0.2863	0.0378	-59814	Continuous
9	1	0.2115	0.4002	1.2911	0.0598	0.001	0.2649	0.0373	-57892	Continuous
10	1	0.2172	0.398	1.2995	0.0545	0.001	0.2467	0.0364	-56366	Continuous

m : Number of periods of divergence-contact
n : Number of admixture pulse during contact
N_1 : Effective size of the Atlantic population in units of N_{ref}
N_2 : Effective size of the Mediterranean population un units of N_{ref}
N : Effective size of the ancestral population in units of N_{ref}
T_c : Duration of contact in units of N_{ref} generations
T_{diff} : Duration of divergence in units of N_{ref} generations
f_1 : Fraction of Atlantic migrating to Mediterranean population
f_2 : Fraction of Mediterranean migrating to Atlantic
generation time : 5 years

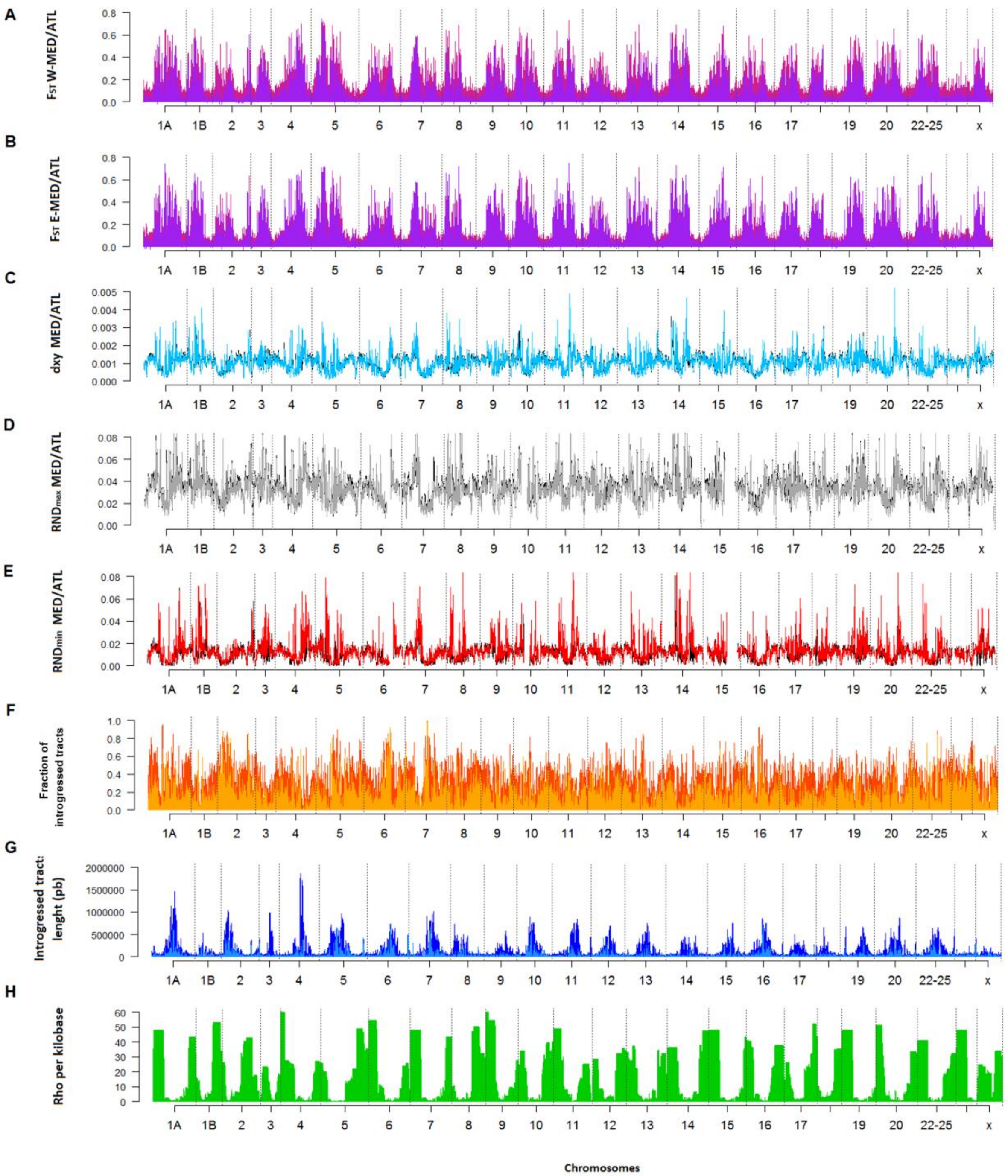

Supplementary Figure 9 – **Comparison of different population genetics statistics calculated in non-overlapping 100 kb windows along the sea bass genome.** Dashed vertical lines represent the limits between chromosomes. Chromosome x does not refer to a sexual chromosome. A. F_{ST} measured between the Atlantic and the contemporary (purple) or ancestral reconstructed (mauve) W-MED population. B. F_{ST} measured between the Atlantic and the present (purple) or ancestral reconstructed (mauve) E-MED population. C. d_{XY} calculated between the Atlantic and the W-MED (black) and E-MED (blue) populations. D. RND_{max} measured between the Atlantic and the W-MED (black) and E-MED (grey) populations. E. RND_{min} measured between the Atlantic and the W-MED (black) and E-MED (red) populations. F. Fraction of introgressed tracts in the W-MED (orange) and E-MED (yellow) populations. G. Average length of introgressed tracts in the W-MED (dark blue) and E-MED (light blue) populations. H. Population-scaled recombination rate (ρ=4$N_e r$ per kb) averaged between Atlantic and Mediterranean populations.

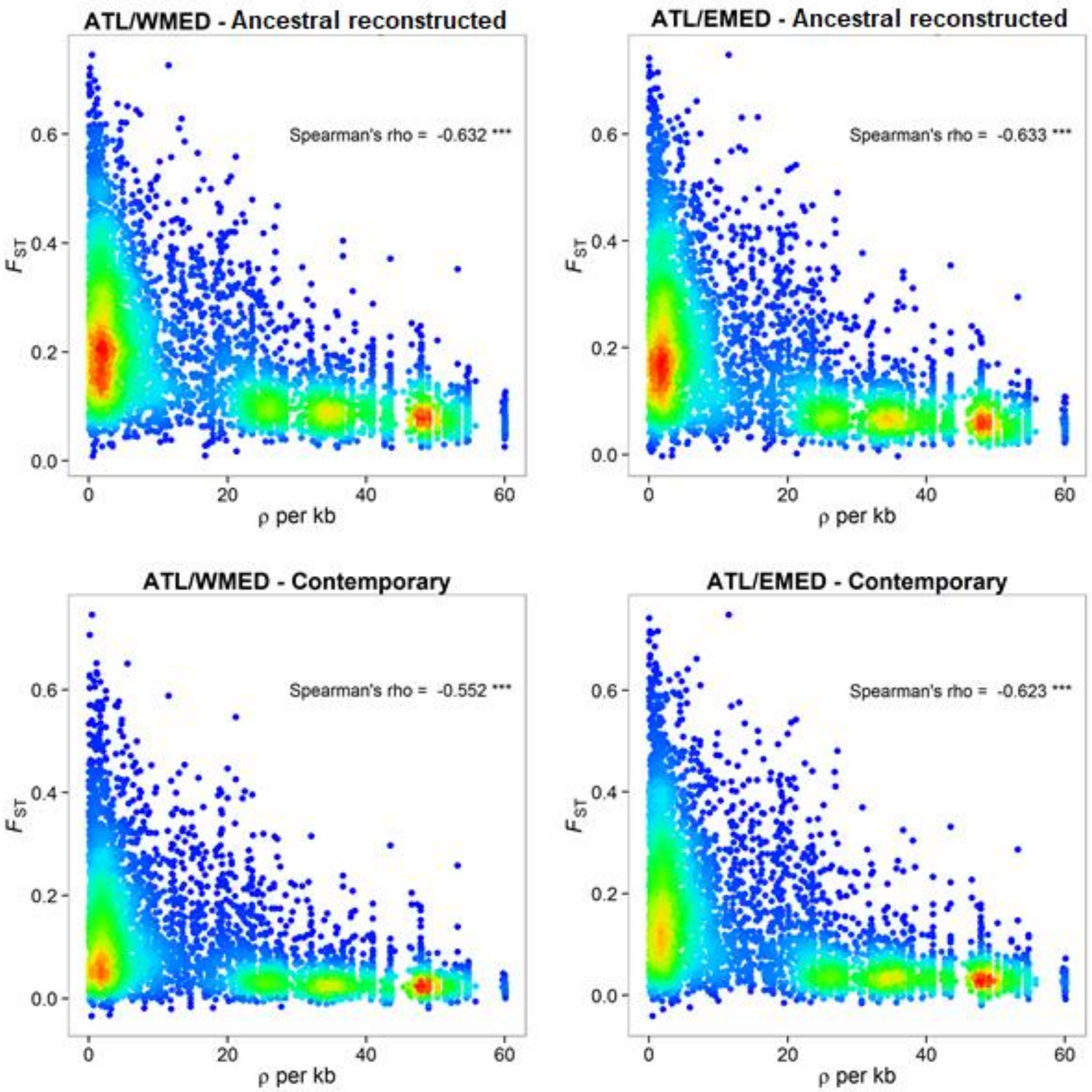

Supplementary Figure 10 – **Relationships between the population-scaled recombination rate (ρ=4$N_e r$ per kb) and genetic differentiation (F_{ST}) calculated in non-overlapping 100 kb windows.** The density of points appears in color scale from low (blue) to high (red) densities.

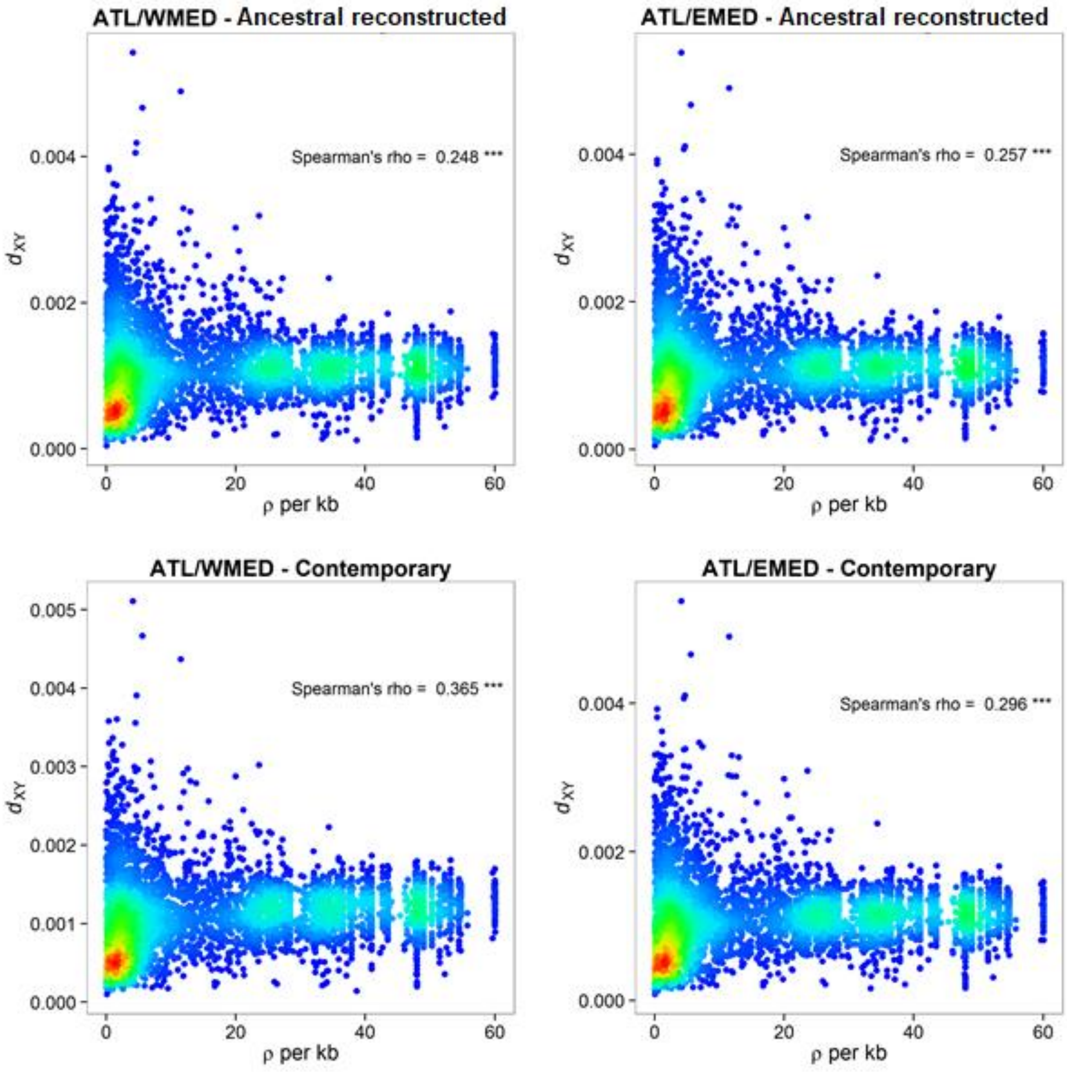

Supplementary Figure 11 – **Relationships between the population-scaled recombination rate (ρ=4$N_e r$ per kb) and nucleotide divergence (d_{XY}) calculated in non-overlapping 100 kb windows.** The density of points appears in color scale from low (blue) to high (red) densities.

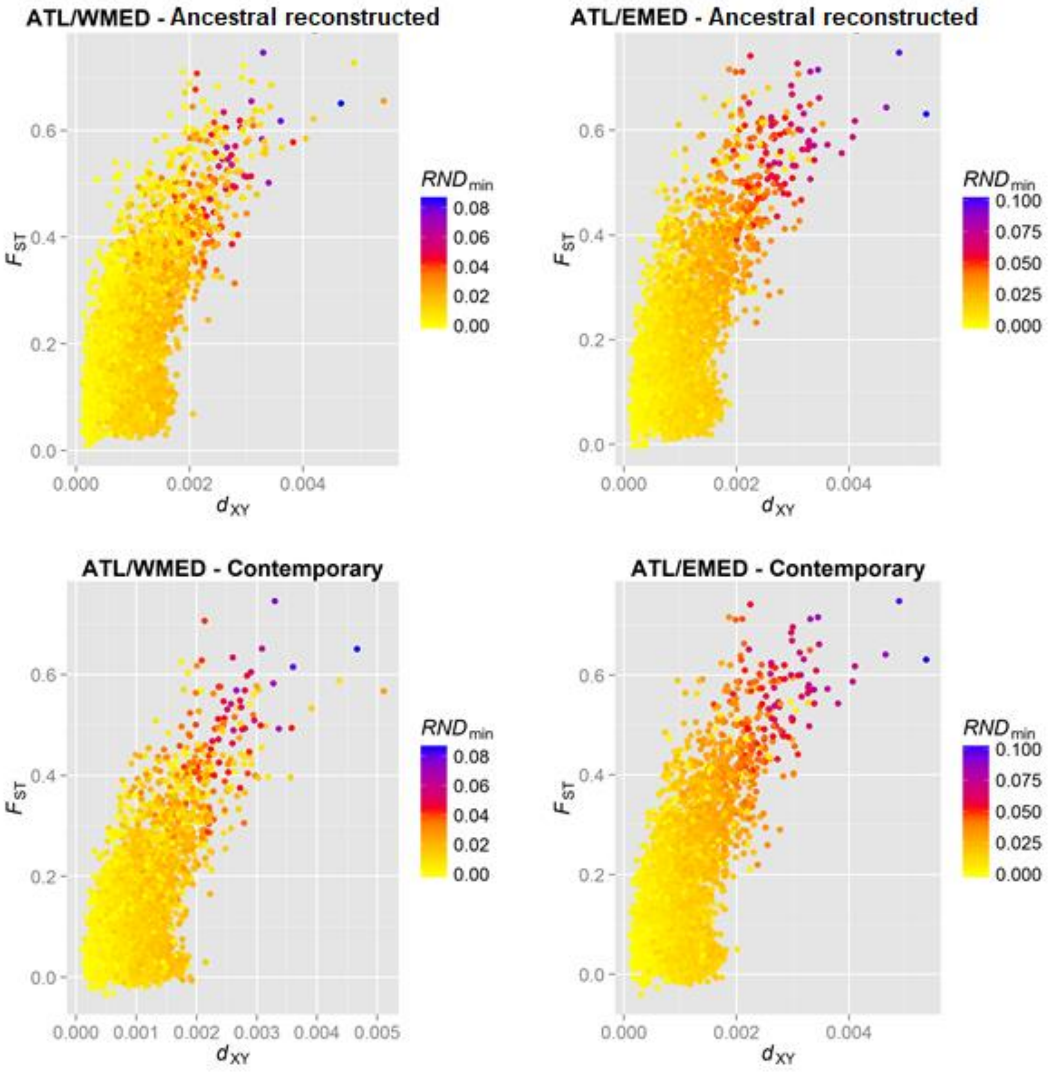

Supplementary Figure 12 – **Relationships between genetic differentiation (F_{ST}) and nucleotide divergence (d_{XY}) calculated in non-overlapping 100 kb windows.** The color scale indicates the value of RND_{min} in the corresponding window from low (yellow) to high (blue) values.

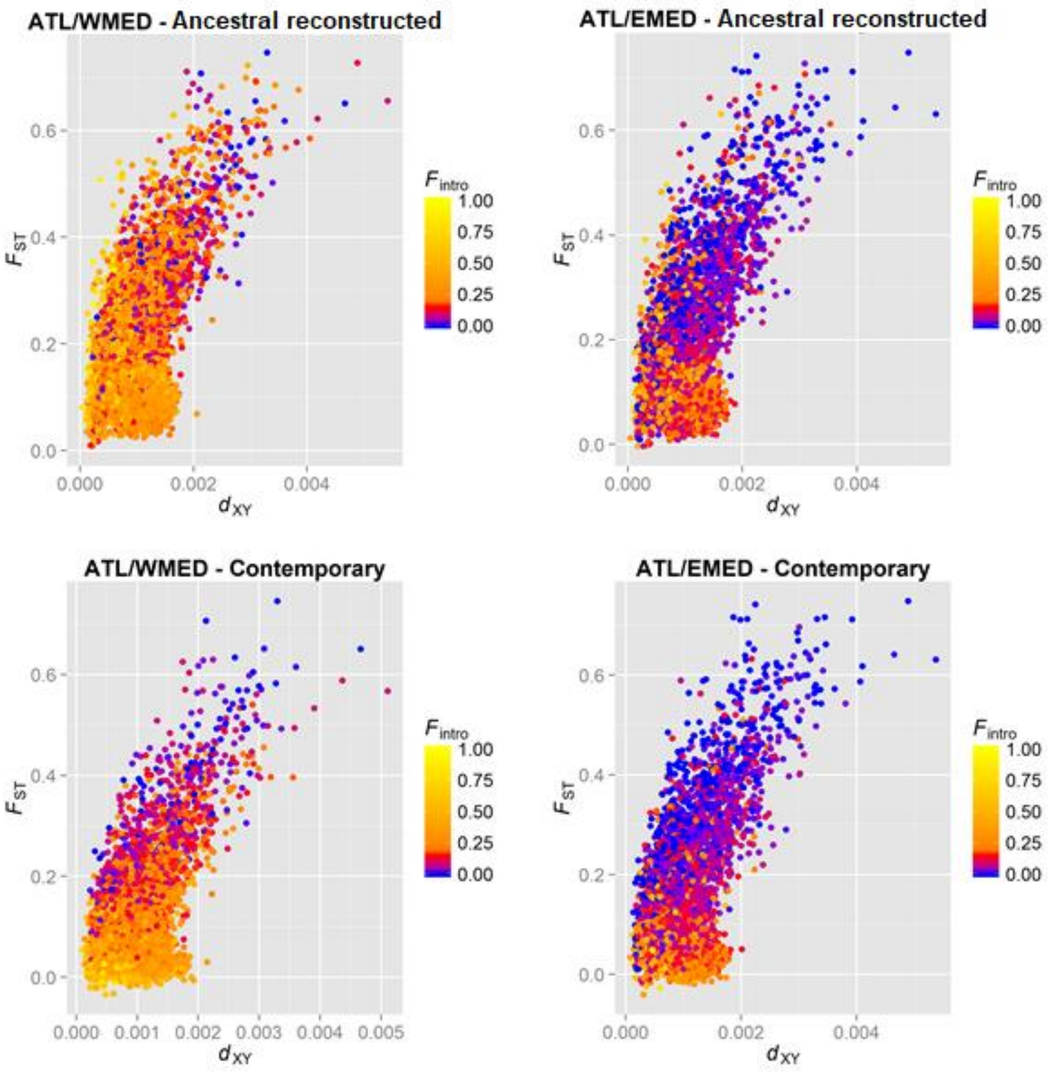

Supplementary Figure 13 – **Relationships between genetic differentiation (F_{ST}) and nucleotide divergence (d_{XY}) calculated in non-overlapping 100 kb windows.** The color scale indicates the frequency of introgression in the corresponding window from low (blue) to high (yellow) frequencies.

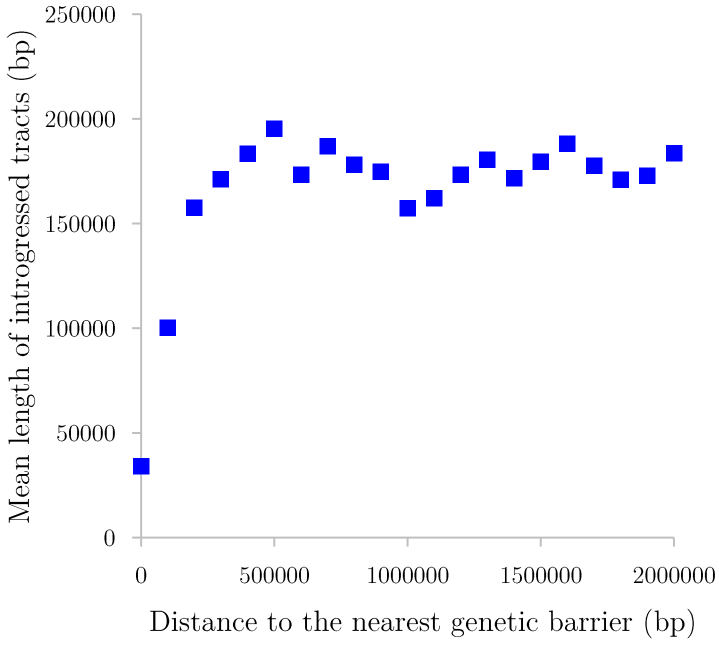

Supplementary Figure 14 – **Relationship between physical distance to the nearest genetic barrier and the mean length of introgressed tracts of Atlantic ancestry found in the W-MED population.** We used the 99th percentile of the distribution of RND_{min} values as a threshold to define outlier genomic regions (100 kb windows) that are highly resistant to introgression and therefore likely contain barrier loci. Each window was used as a reference position from which we calculated the mean length of Atlantic migrant tracts at increasing physical distances. Measures were finally combined and averaged across outlier regions.

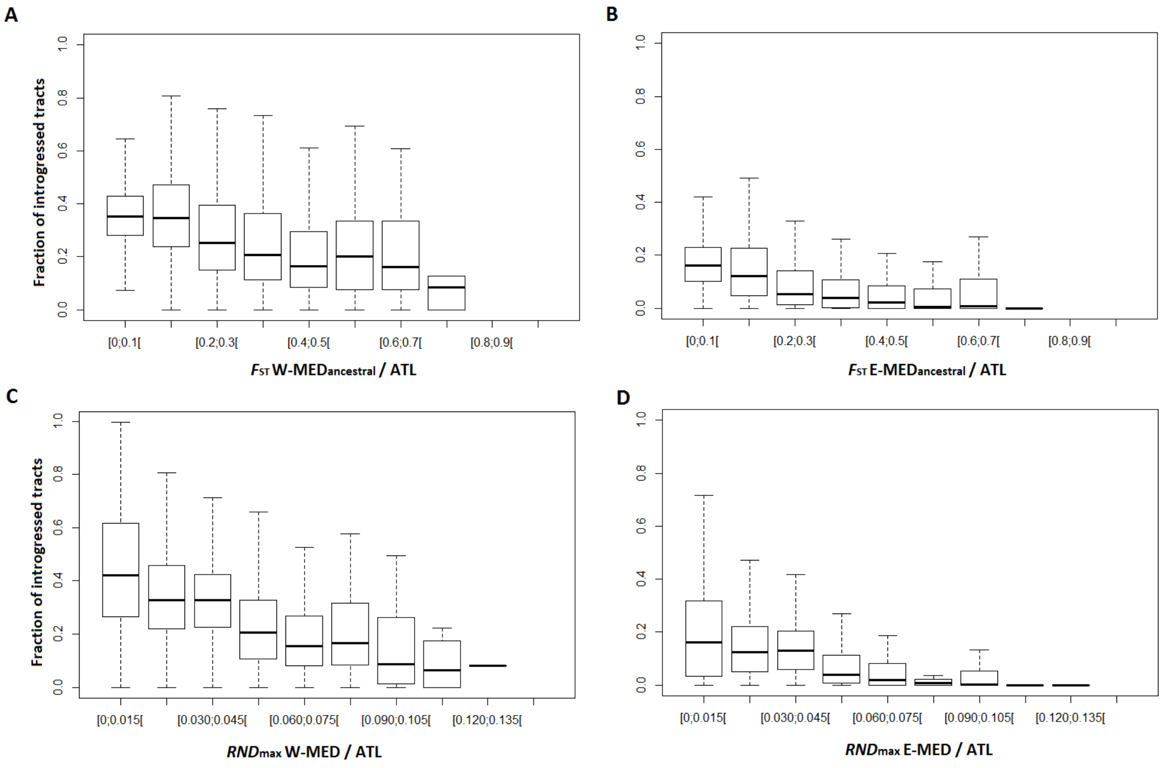

Supplementary Figure 15 – **Fraction of introgressed tracts for different levels of ancestral genetic differentiation and allelic divergence measured with RND_{max}.** A. Introgression as a function of ancestral F_{ST} measured between the Atlantic and the reconstructed ancestral W-MED or E-MED (B.) population. C. Introgression as a function of RND_{max} measured between the Atlantic and W-MED or E-MED (D.) population. Each box represents the lower and upper quartiles and the median of introgression frequency values. The negative correlation between the fraction of introgressed tracts and ancestral F_{ST} is not a methodological artifact due to the removal of introgressed tracts from contemporary genomes. This procedure would on the contrary tend to overestimate the ancestral F_{ST} in highly introgressed regions, where the reconstructed ancestral diversity of the Mediterranean population may be downwardly biased.

Supplementary References

1. Tine, M. *et al.* European sea bass genome and its variation provide insights into adaptation to euryhalinity and speciation. *Nature Communications* **5**, 5770 (2014).
2. Li, H. Aligning sequence reads, clone sequences and assembly contigs with BWA-MEM. *arXiv preprint arXiv:1303.3997* (2013).
3. Danecek, P. *et al.* The variant call format and VCFtools. *Bioinformatics* **27**, 2156–2158 (2011).
4. Jombart, T. adegenet: a R package for the multivariate analysis of genetic markers. *Bioinformatics* **24**, 1403–1405 (2008).
5. Lawson, D. J., Hellenthal, G., Myers, S. & Falush, D. Inference of Population Structure using Dense Haplotype Data. *PLOS Genet* **8**(1), e1002453 (2012).
6. Pool, J. E. & Nielsen, R. Inference of Historical Changes in Migration Rate From the Lengths of Migrant Tracts. *Genetics* **181**, 711–719 (2009).
7. Liang, M. & Nielsen, R. The Lengths of Admixture Tracts. *Genetics* **197**, 953–967 (2014).
8. Racimo, F., Sankararaman, S., Nielsen, R. & Huerta-Sánchez, E. Evidence for archaic adaptive introgression in humans. *Nat Rev Genet* **16**, 359–371 (2015).
9. Harris, K. & Nielsen, R. Inferring Demographic History from a Spectrum of Shared Haplotype Lengths. *PLOS Genet* **9**(6), e1003521 (2013).
